# Metal Nanocomposites as Biosensors for Biological Fluids Analysis

**DOI:** 10.3390/ma18081809

**Published:** 2025-04-15

**Authors:** Dan Chicea, Alexandra Nicolae-Maranciuc

**Affiliations:** 1 Research Center for Complex Physical Systems, Faculty of Sciences, Lucian Blaga University of Sibiu, 550012 Sibiu, Romania; 2 Institute for Interdisciplinary Studies and Research (ISCI), Lucian Blaga University of Sibiu, 550024 Sibiu, Romania

**Keywords:** metal nanocomposite, biological fluids, sensors, microfluidic devices

## Abstract

Metal nanocomposites are rapidly emerging as a powerful platform for biosensing applications, particularly in the analysis of biological fluids. This review paper examines the recent advancements in the development and application of metal nanocomposites as biosensors for detecting various analytes in complex biological matrices such as blood, serum, urine, and saliva. We discuss the unique physicochemical properties of metal nanocomposites, including their high surface area, enhanced conductivity, and tunable optical and electrochemical characteristics, which contribute to their superior sensing capabilities. The review will cover various fabrication techniques, focusing on their impact on the sensitivity, selectivity, and stability of the resulting biosensors. Furthermore, we will analyze the diverse applications of these biosensors in the detection of disease biomarkers, environmental toxins, and therapeutic drugs within biological fluids. Finally, we will address the current challenges and future perspectives of this field, highlighting the potential for improved diagnostic tools and personalized medicine through the continued development of advanced metal nanocomposite-based biosensors.

## 1. Introduction

Biosensors are analytical devices that combine a biological component with a physicochemical detector to measure specific substances. These devices convert biological reactions into measurable electrical signals.

Biosensors are extensively used in environmental monitoring, medical diagnostics, and food safety due to their high specificity, sensitivity, and rapid response. By integrating biological and electronic elements, they serve as crucial tools in modern technology for efficiently detecting various biochemical changes.

In the context of biological fluid analysis, biosensors play a pivotal role in detecting and quantifying analytes such as glucose, lactate, electrolytes, and various biomarkers. Electrochemical biosensors have been developed for non-invasive, real-time monitoring of disease-indicating biomarkers in biofluids like tears, saliva, breath, urine, and sweat. These sensors offer high sensitivity, selectivity, and cost-effectiveness, making them promising tools for individualized health monitoring [1].

The integration of biosensors into point-of-care (POC) settings has the potential to revolutionize patient care by providing rapid, quantitative diagnostic information without the need for centralized laboratory assays. This approach can lead to better patient outcomes and a reduced burden on the healthcare system [2].

Metal nanocomposites are hybrid materials that integrate metal nanoparticles with other substances, such as polymers or ceramics, to create a composite with enhanced or novel properties. These materials combine the advantageous characteristics of their individual components, resulting in unique combinations of mechanical, electrical, optical, and thermal properties [3].

One of the key attributes of metal nanocomposites is their ability to exhibit unusual property combinations and unique design possibilities. For instance, metal–polymer nanocomposites combine the superior plasmonic, electrical, and thermal properties of metals with the good elasticity and manufacturability of polymers, making them promising candidates for conductive fillers and coating applications [4].

In biosensing, metal nanocomposites provide substantial benefits. Their unique properties, such as enhanced electrical conductivity and catalytic activity, can improve the sensitivity and selectivity of biosensors. For example, incorporating metal nanoparticles into a polymer matrix can create a nanocomposite with tailored electrical properties suitable for detecting specific biological analytes [5].

Moreover, the tunable nature of metal nanocomposites allows the optimization of their properties to suit specific applications. By adjusting factors such as the type of metal used, particle size, and the composition of the surrounding matrix, there can be designed nanocomposites with desired characteristics for targeted biosensing applications [6].

Microfluidic technology can be integrated into biosensors, thus offering significant advantages for the detection of emerging biomarkers in biological fluids. Microfluidic biosensors are designed to determine the composition and concentration of biomarkers in samples that are separated or processed using microfluidic systems [7]. One of the primary benefits of microfluidic biosensors is their ability to handle small sample volumes, which is particularly advantageous when dealing with limited or precious biological fluids. Additionally, these systems can provide rapid analysis times, which is crucial for timely diagnosis and monitoring [8].

Metal nanocomposites enhance biosensors through five key advantages. Gold/silver nanoparticles (AuNPs/AgNPs) increase detection limits up to 1000× via plasmonic effects and a high surface area, thus boosting sensitivity [9]. Another advantage lays in multifunctional detection, enabling optical, electrochemical, and magnetic sensing in a single platform. Precision targeting is another advantage. The surface-modified nanoparticles selectively bind biomarkers while resisting interference [10].

The metal nanocomposites present enhanced durability and, therefore, can withstand harsh conditions while maintaining stable performance over time [11]. Moreover, metal nanocomposites facilitate the development of compact, flexible biosensors ideal for wearable health monitoring applications [12]. These advancements are transforming medical diagnostics and environmental monitoring, particularly for POC applications where reliability and sensitivity are critical. Recent breakthroughs include sweat-analysis patches and ultra-sensitive pathogen detectors, demonstrating the technology’s potential for real-world implementation.

The focus on emerging biomarkers is driven by the need for the early detection and monitoring of diseases. Emerging biomarkers can provide critical information about disease states, enabling earlier interventions and personalized treatment strategies. Microfluidic biosensors have emerged as powerful tools for detecting these biomarkers in body fluids, providing enhanced sensitivity, specificity, and rapid analysis.

## 2. Fundamentals of Metal Nanocomposites

Sensor development for the precise detection of biological molecules and chemical compounds has become essential for early crucial diagnosis and prevention [13]. Recently, nanotechnology has become more and more present in our daily lives. Based on very small particles with specific dimensions, nanomaterials can be found in various forms in vast applications [14]. The integration of nanotechnology in processes around us enhances our life quality due to the unique properties and functions offered by the materials. Besides the qualitative aspects, the economic and industrial impact is greatly enhanced by the integration of these alternative solutions offered by nanoscience [15].

Nanocomposites can be defined as multiphase or hybrid materials combined together to improve the final properties of the biomaterial proposed [16,17,18]. Nanocomposites show innovative properties compared to their bulk sizes, especially due to the high surface-to-volume ratio and to their increased reactivity [19]. If we consider a mass m of bulk materials, e.g., Ag having the density of *ρ* = 10,503 kg/m^3^ and turning into more spheres with lower and lower radii, the total surface exhibited by the same mass of material will increase as the radius of the particle decreases. If *N* is the number of spherical particles that can be made from a mass m of bulk material and *S* is the area of the total surface of the spheres, Equation (1) describes these variables.

(1) $$N=\frac{3m}{4\pi \rho R^{3}};S=4\pi R^{2}N=\frac{3m}{\rho }\frac{1}{R}$$

**Figure 1:**
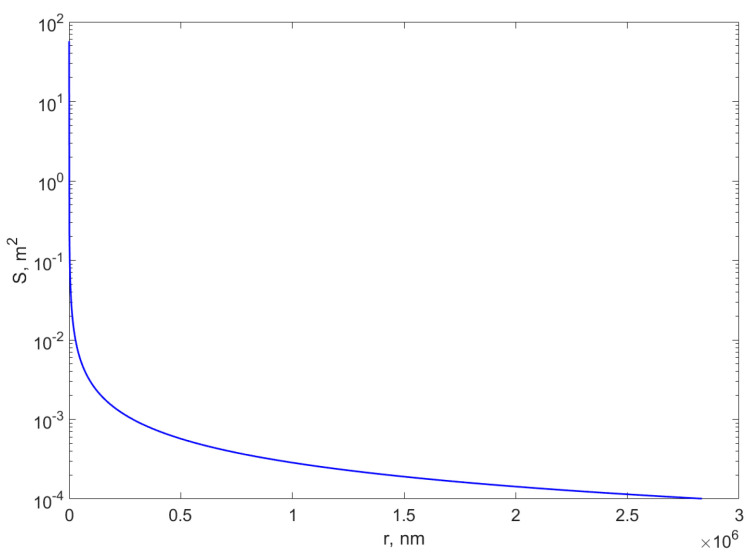
The total surface exhibited by a mass m = 1 g of Ag for different radii *R* values of the spheres it is turned into, according with Equation (1), from the radius of one single sphere down to 5 nm.

Figure 1 illustrates the total surface exhibited by a mass *m* = 1 g of Ag for the different radii *R* values of the spheres it is turned into, according with Equation (1).

The improved optical and electrical properties of metal particles offered by the nanometric size are mentioned in many reports [20,21]. Once the nanometric size is achieved, the surface of each small nanoparticle will be able to interact with further compounds. Compared to the bulky dimension, when only one or a few surfaces will be in contact with future molecules, the huge number of surfaces offered by each nanoparticle will increase the final reactivity of the material. In optical biosensors, AuNPs are used to improve the sensing properties since they show strong absorption in visible and infrared regions and can generate a strong electric field on the particle’s surfaces [22]. Compared to Au, AgNPs also are known for their high sensitivity properties. AgNPs possess better biosensing properties due to their higher dielectric constant [22,23]. In a recent study, Patel et al. [24] successfully reported the use of Ag NPs in the development of an electrochemical biosensor for Shiga toxin detection. By comparison with AuNPs, AgNPs-based sensors are cheaper and can be modified easier during the fabrication method; therefore, the development of such biosensors for the detection of biomolecules is a promising approach. The group successfully conjugated AgNPs with the specific antibody for the toxin, reaching an impressive detection limit of 2 ng/mL, comparable with classical detection techniques, such as ELISA or PCR, which could require high amounts of expensive reagents and high periods of time [24]. Another metal studied for its increased surface properties due to the nano scale is copper (Cu), used mostly in its oxidative form as copper oxide (CuO). Recent studies are targeting non-enzymatic biosensors for biomarkers detection based on metals, nanoparticles, or nanocomposites. For instance, Magar et al. [25] developed an impedimetric biosensor based on CuO/Co_3_O_4_ metal oxide with multi-walled carbon nanotubes in its composition for fast urea detection with high stability and direct application. With high electrochemical performance, the biosensor was designed based on disposable screen-printed electrodes that were functionalized with the nanocomposite. The physical–chemical characterization successfully proved the nanocomposite fabrication, while the direct sensing of urea in real samples was accomplished with high sensitivity in a range from 10^−12^ M to 10^−2^ M and with a limit detection of 0.223 pM [25]. Another recent study was reported by Zohaa et al. [26], who designed an electrochemical sensor based on CuO nanoparticles integrated into mesoporous MCM-41 silica for the non-enzymatic detection of glucose. Due to high mechanical and thermal stability of silica, nanocomposites based on metal nanoparticles and mesoporous silica are widely used in bionanotechnology. Moreover, the transport of biomolecules is improved due to the increased surface area. The study results demonstrated the formation of metal nanoparticles at a nanoscale level, with superior electrocatalytic properties with an electrochemical response of glucose from 83 μM to 1.5 mM [26].

This increased chemical reactivity of nanoparticles used in biosensing applications can also be considered a drawback in some circumstances. Due to their small dimensions, metal nanoparticles can easily interact with cells, tissues, or simple biomolecules, which might absorb them and cause a toxic response of the organism [27]. Their integration in nanocomposites and in a stable matrix will decrease these possible side effects in the long-term. In addition, the combination of various materials enhances the specific characteristics of each composite regarding the application it was designed for. A nanocomposite is a mix of a minimum of two materials in which one of the phases is found in dimensions under 100 nm [28]. Depending on the constituents, the phases from them can be a mixture of inorganic–inorganic, inorganic–organic or organic–organic materials [29]. The matrix and reinforcement phases found in a composite material are the main components of these types of materials. The role of the matrix is extremely important since it holds together the entire system in the desired design [30]. The reinforcement phase must also accomplish some criteria since the interaction in a composite material structure should be assured after implantation. Reinforcement materials should be compatible and chemically stable so that the physical–chemical properties of the composite are at maximum activity levels [30,31].

### 2.1. Nanocomposites Based on Metal Nanoparticles: Classification and Properties

**Figure 2:**
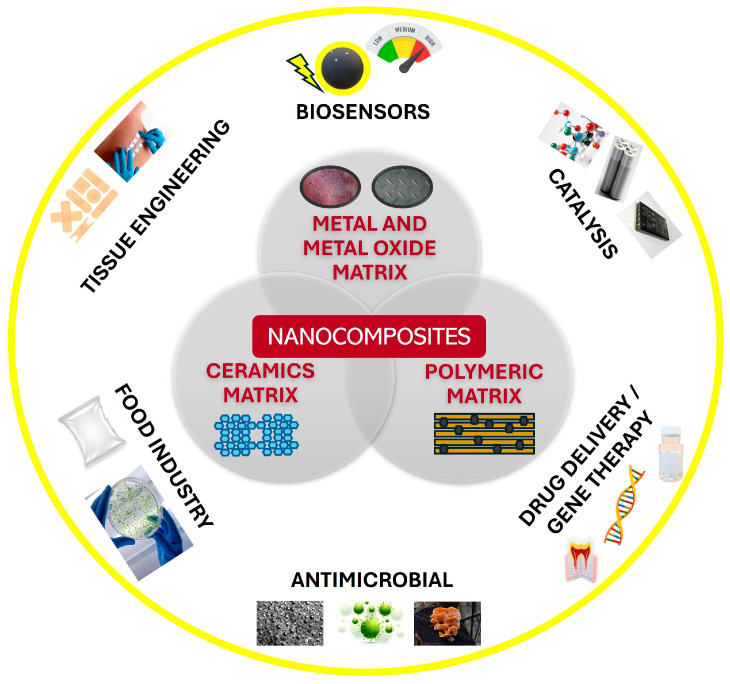
Main classification and applications of nanocomposites in bionanotechnology.

The main classification for nanocomposites is based on their constituents. Therefore, the nanocomposites can be mixtures of metal matrix composites, polymer matrix composites, and ceramic matrix composites, according to their matrix with multiple applications, as is described in Figure 2 [32].

Metal and metal-oxide composites are among the most studied materials since they are highly versatile and allow the development of medical devices for a precise detection. Polymers typically have limited strength, thermal resistance, and conductivity. To expand their uses, metal–polymer nanocomposites with improved optical and electrical properties were developed [33].

Metal nanoparticles, known also as plasmonic nanoparticles [34], have special properties used in fluid detection. They are known as good optical alternatives, they possess electrical, mechanical, and magnetic properties; therefore, they are suitable for electronic device fabrication. Silver [35], gold [36], or palladium [37], known as noble metals [38], together with nanotechnology, represent a promising strategy in designing smart tools to solve medical conditions. Among them, Ag and Au are considered an optimal choice in nanomaterials fabrication since they are versatile, exhibit excellent optical and catalytical properties, and can be considered an effective target in targeted applications [39]. Additionally, their most important advantage is the plasmonic activity [34,40] which encourages their use as photonics in biosensors applications for fluid detections. Their ability to manipulate light, together with their biomedical purposes, creates a perfect synergy for energy manipulation and detection applications. Through exposure to electromagnetic radiation, metal nanoparticles show an optical phenomenon known as surface plasmon resonance (SPR) or localized surface plasmon resonance (LSPR) [41,42]. The most frequent electromagnetic radiation absorption is from the ultraviolet up to the near-infrared spectrum, depending on the metal subjected to light. Under these conditions, the electrons from metal nanoparticles begin to oscillate to a resonant frequency based on the positive ions network [43]. This phenomenon leads to a strong absorption of the light emitted or to the scattering of the radiation [44].

Biosensors based on metallic nanoparticles can be found in different combinations depending on the number of metals involved: mono-, bi-, tri- and quadrometallic nanoparticles [45]. Monometallic nanoparticles are composed of a single metal, which should offer the plasmonic characteristics for the system created by also assuring a biomedical application based on high biocompatibility. These types of materials are based on a single type, for example Ag, Au, Ti, Al, Zn, Bi, or Si [46], which can assure various effects. Most studies are related to the antimicrobial activity; however, their integration in optical or electronic devices can assure a precise detection of fluids [45]. Based on the type of metal and on the crystalline structure or oxidation state, the metal can be processed by different routes [47]. Metal oxides, such as Ag_2_O, ZnO, TiO_2_, CuO, MgO, Al_2_O_3_, and Fe_3_O_4_, have also demonstrated their impact in biological applications. They are used as antimicrobial/antioxidant agents [48], in drug delivery systems [49], biosensors [50,51,52], targeted therapies using medical imaging [53], in the food industry [54], but also in optical and electronic devices [55]. Both metallic and metal oxide particles can be used in various applications such as medical, environmental, or agricultural biosensors [45]. Due to their large surface-area-to-volume ratio and the reactive electronic structure, the number of studies containing monometallic nanoparticles has increased lately. Paszkiewicz et al. [56] synthesized silver and copper monometallic nanoparticles using a classical technique of chemical reduction for antimicrobial applications. The results showed a higher efficiency of silver nanoparticles compared to copper for both *Escherichia coli* and *Staphylococcus* aureus strains [56]. Mahajan et al. [57] fabricated monodisperse magnetic γ-Fe_2_O_3_ nanoparticles through a chemical solvothermal synthesis for target therapies using magnetic resonance imaging. More recently, Nyabadza et al. [58] described in a review paper the pulsed laser ablation in liquid physical techniques to fabricate these types of monometallic nanoparticles. The technique is based on the bombardment of a solid target under a liquid, using lasers to produce colloids. The monometallic nanoparticles provide enhanced electric properties; therefore, they can be used in flexible electronics designs [58,59].

Bimetallic nanoparticles are composed of two different metals by synthesis, based on the differences between two metallic particle architectures. The main advantage of bimetallic particles is the optimization of the plasmonic absorption bans offered by the two metals, which enhance the biosensing capacity of the material. Through the process of bimetallization, the catalytic properties are improved; therefore, the charge transfer is increased. A higher energy is released in the case of bimetallic particles compared to monometallic ones; therefore, the surface and adsorption properties are improved [16,60,61]. Based on the chemical interactions, the atom distribution in such complexes can lead to a core–shell structure or to an alloy. As mentioned by multiple papers, the addition of the second metal will increase the catalytic and selectivity properties of the sensor [45,62,63]. The synthesis parameters are essential in bimetallic sensor development since the structure and miscibility of the particles can be modified by the reduction reaction, which is generally used for plasmonic particles fabrication [16]. The control of the reaction based on the two metals can be obtained by assuring certain sizes, shapes, or surface-modifications. However, there are concerns addressed to bimetallic materials regarding their chemical stability during the fabrication process and their possible toxic effect as well. Regarding the synthesis, the particle sizes and final nanoparticle shape can be affected if the chemical reaction cannot be controlled. Whether the metals are present at the same time in the reaction appears to be an important parameter, influencing the product formation. Simultaneous presence can lead to an alloy, unlike the sequential addition and reduction of each metal. In the case of Au and Ag, the second possibility would lead to a core–shell form. However, in terms of practicality, the possibility to control the redox reaction at 100% is quite small; therefore, the stability of the complex could be decreased [64]. It is worth mentioning that once two metals are combined, the possible toxicological effect in the long-term can increase. Compared to the monometallic materials, the addition of another metal can increase the quantity of the ions released at the area of the body where the biosensor is implemented if the proper integration of metal particles in a biocompatible matrix is not achieved. Another approach sustained lately by researchers is the use of polymers or ceramics to increase biocompatibility and biodegradability.

Bimetallic nanocomposites integrate metal particles into a ceramic or polymeric matrix [65], depending on the final application. These composites are multifunctional; they usually combine the plasmonic response of nanoparticles with the mechanical or elastic properties of the matrix in which they are integrated. Covered by a shell or added into thin films, nanoparticles can act while the risks associated with the utilization of the biosensor decrease. The catalytical, electrical, optical properties are improved while maintaining the nano size, and the polymeric matrix offers comfort and support for the patient. The agglomeration of nanoparticles will decrease, since the synthesis is usually controlled in this regard. Recent studies report higher surface-enhanced Raman scattering, and electronic, optic, magnetic, and catalytic properties for bimetallic nanocomposites; therefore, their use in biosensor fabrication is increasing lately [66,67,68]. In a recent paper, Zhong et al. [69] synthesized PtCu_3_ nanocages stabilized with poly(ethylene glycol) (PEG) for cancer therapy. The system was able to generate ROS once it was under ultrasound irradiation, and was able to accelerate GSH (glutathione peroxidase), which can help to decrease tumor cells activity. The biological results also showed that the system is able to alter the cancer cells, while the normal cells are degraded in a small percentage [69]. Another study involving bimetallic nanoparticles and carbon-based material was conducted by Das et al. [70]. A nanocomposite consisting of Au/Pd embedded in a matrix of polydopamine functionalized with graphene was subjected to physical, chemical, and biological characterization. Once the formation of nanoparticles was confirmed by UV-VIS, XRD, and TEM analysis, the biocompatibility tests performed on L292 cell lines proved no cytotoxicity for 48 h at concentrations lower than 150 μg mL^−1^. The nanocomposites also showed important photothermal activity for cancer cells even in a lower concentration. The irradiation was performed with NIR laser 915 nm at different times, and the maximum 55 °C temperature achieved proved to be sufficient for the ablation of tumor cells [70].

Multimetallic nanoparticles, known as tri or quadrometallic particles, are characterized by tunable and stronger optical, electric, and catalytic properties compared to the mono and bimetallic particles. The presence of three or four metals in the same material leads to a synergistic effect since the electronic reactivity, different morphologies, surfaces, or chemical structures are combined. In the case of biosensors, these associations of metal elements increase the activity of the device which can be applied for specific applications. However, when comparing them to mono or bimetallic nanoparticles, the possible toxic effect should be addressed in long-term studies since possible harmful effects can appear at the surrounding tissues or even at the implantation area. To discuss this issue, a sub-section regarding the challenges for multimetallic nanoparticles is addressed in this section. Various studies have reported the use of tri or quadrometallic nanoparticles for medical applications. Ye et al. [71] synthesized a promising catalyst, Cu/Au/Pt trimetallic nanoparticles, using a co-reduction method. The proposed material was designed as a biosensor for glucose detection and as a theragnostic alternative for cancer detection. The sensible and low detection of 25 μM obtained for glucose detection was considered a great strategy by comparing to other colorimetric methods in which the detection was achieved with greater difficulty [71]. Yadav et al. [72] showed that trimetallic Au/Pt/Ag nanofluid has a stronger bactericidal effect for a large number of bacteria strains compared to the mono and bimetallic fluids synthesized [72]. The idea was confirmed by other studies as well, in which the enhanced antibacterial properties were proved once the association of more metals was approached [73,74,75]. A comprehensive review paper, in which the activity of trimetallic nanoparticles compared to simpler materials was reported, was conducted by Zhang and Toshima [76]. The paper compared the catalytic activity of monometallic, bimetallic, and trimetallic nanoparticles for glucose oxidation. Once the number of metals increases, the degree of catalytic activity and selectivity also increases, leading to higher activity for glucose oxidation. Many factors, such as geometric effect, electronic structure alteration, or change transfer, were assumed to be involved in this increased catalytical activity of multimetallic nanoparticles [76]. Table 1 shows an overview of different metal-based nanocomposites using mono or multimetallic nanoparticles for fluids detection.

**Challenges in the Biomedical Application of Multimetallic Nanocomposites for Biosensing**

Together with the considerable scientific interest owing to their superior detection capabilities, important questions persist regarding their potential long-term toxicity and limited biodegradability, which must be resolved for successful clinical translation.

The distinctive physicochemical characteristics of multimetallic nanoparticles, arising from the synergistic combination of different metallic elements, present both opportunities and challenges for biological applications. While these hybrid nanostructures demonstrate enhanced performance in sensing applications, their complex interactions with living systems may lead to undesirable biological effects. Current investigations have revealed that certain multimetallic NP formulations can trigger cellular damage through mechanisms, including membrane disruption, reactive oxygen species generation, and activation of inflammatory pathways. The nature and extent of these effects appear dependent on multiple parameters, including, but not limited to the following: elemental composition, particle dimensions, morphological characteristics, and surface functionalization. For example, studies examining gold–silver bimetallic systems have emphasized the necessity for rigorous preclinical evaluation to establish safety profiles for diagnostic applications [92].

The limited biodegradation potential of metallic nanocomposites represents another significant barrier to their widespread clinical adoption. Unlike biologically-derived materials, most metal-based nanostructures demonstrate remarkable persistence in physiological conditions, potentially leading to bioaccumulation in various organ systems [93]. This prolonged retention may not only amplify toxicity risks but also complicate the body’s natural clearance mechanisms. In response to these challenges, researchers have developed innovative hybrid architectures that combine metallic NPs with enzymatically-degradable polymeric components, creating composite systems designed for gradual breakdown and elimination.

Current strategies to address these safety concerns focus on three primary directions. The first direction involves the application of biocompatible surface modifications, such as PEGylation or protein coronas, which can significantly improve nanoparticle stability while reducing nonspecific interactions with biological components. The second direction focuses on the development of nanocomposite scaffolds using biodegradable polymers (e.g., polylactic acid, chitosan), which enable the controlled release and subsequent clearance of metallic components through natural metabolic pathways. Morphological optimization, which is the third direction, implies a precise control over nanoparticle geometry and dimensional parameters that allow for the tuning of biodistribution patterns and enhancement of renal clearance efficiency.

### 2.2. Metal Nanoparticles Syntheses

Nanocomposites used in biosensing applications for biological fluids are usually fabricated in many steps. The first step is represented by the metal nanoparticle synthesis at a nanoscale level proved by physical–chemical characterization. Since dimension is the main factor for optimal optical and electrical properties, the confirmation of nano sizes in a stable suspension without agglomeration is mandatory for each performed synthesis. Many synthesis routes have been described lately in the literature for these plasmonic nanoparticle fabrications [94,95,96], as will be further explained in this review. In the second step, the biosensor is obtained under various fabrication approaches by incorporating the plasmonic nanoparticles into a stable, comfortable, yet strong enough matrix.

The synthesis methods proposed for metal nanoparticles [97,98] can be classified according to the classical top-down [99] and bottom-up [100] methods. The principles are similar in all synthesis methods, by reducing the metal salt into a neutral stable chemical form. The bottom-up methods assume the formation of nanoparticles by the self-assembly or controlled growth of molecules [97], while the top-down approach is based on the transformation of a bulk material into nano-sized particles under physical conditions [101].

**Figure 3:**
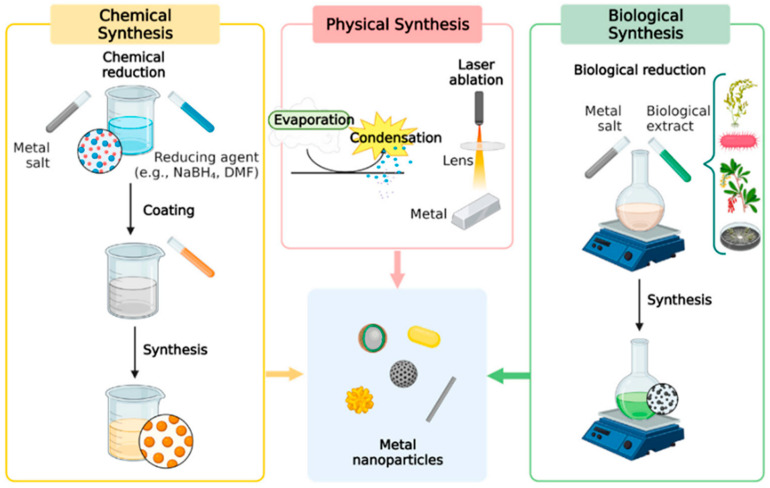
Synthesis methods for metallic nanoparticles in biosensors. Reprinted from an open-access article [39].

Metal nanoparticles and metal-based nanocomposite syntheses can be also classified according to the process chosen in the laboratory. Therefore, the plasmonic metal nanoparticles used in detection applications can be designed using a chemical, physical, or biological synthesis, known also as a green synthesis, as can be observed in Figure 3.

Physical synthesis is an approach in which electromagnetic radiation [102], heat [103], or plasma [104] is used to transform the bulk material into powder and then into nanoparticles. Compared to other methods, the physical ones are applied less since they are costly and strenuous [105]. Sputtering [106], physical vapor deposition [107], laser ablation [108], or arc discharge [109] are physical synthesis methods in which metal particles can be fabricated. However, since these types of methods involve time, expensive equipment, reagents, and specialized researchers, chemical methods have gained more attention lately.

Chemical reduction and green synthesis are similar approaches performed to obtain nanoparticles. The chemical routes use chemical reagents; therefore, the protocols are usually established and the reproducibility is assured at specific concentrations. The weaknesses of this approach, based on the limitations of the reducing agents and the huge number of studies performed in the literature, led to the development of green synthesis, a natural chemical synthesis in which compounds found in nature are used to reduce the metal salt. However, in the case of green synthesis, some issues can be addressed. Unlike chemical synthesis, this reaction has lower stability, leading to assumptions about the underlying reduction process. The high number of compounds found in a plant, for example, can react uncontrollably with the metal salt, and the production of secondary complexes can appear. Also, the sensitivity of the reaction is bigger; therefore, the reproducibility of the synthesis is usually lower compared to the classical chemical routes. The freshness of the plants or microorganisms used as reducing agents is also mandatory. However, this field of green synthesis has gained researchers’ attention due to the high number of possibilities regarding plants. Even if in the last years a lot of studies have appeared, new biological or natural compounds can be tested to design innovative metal nanoparticles with plasmonic properties for biosensing applications.

Chemical syntheses are the most used approaches in terms of the synthesis of metal nanoparticles [110]. The chemical process is usually based on three components: a precursor, a reducing agent (for example, NaBH_4_), and a capping/stabilizing agent, and depends on the solvent characteristics [15]. Besides concentration, time, and temperature, the pH can determine the final shape and dimensions of the particles synthesized. Depending on the final application, the size and shape of metal nanoparticles can be easily adjusted during the synthesis. Sol–gel [111], hydrothermal methods [112], microemulsion [113], or electrochemical approaches [114] are chemical synthesis routes studied lately for metal nanoparticle fabrication. Chemical reduction, the main synthesis method for metallic nanoparticle fabrication, is based on the reduction of a metal precursor into stable nanoparticles using reducing and stabilizing agents. It is a cost-effective and high yield process, is affordable, easy to apply in laboratories, and most importantly, is controllable.

Plant-mediated green synthesis is a cost-effective, eco-friendly, non-toxic method to obtain nanocomposites which can be further integrated into an electronic device as biosensors [115,116]. Based on medicinal or antioxidant plants, the reaction is a classical chemical reduction; however, the reducing agents are found in the chemical composition of the plant, bacteria, or viruses [117]. Plant extracts are obtained through different processes, such as hot extraction or cold extraction, which are able to maintain intact the chemical compounds used further for the reduction reaction [46]. Plants have a history of use in material synthesis. More recently, Parveen et al. employed *Sanvitalia procumbens* in a green synthesis approach to obtain zirconium-doped cerium oxide nanoparticles [118]. The proposed nanocomposites proved to have an UV-VIS absorption increase while the concentration of cerium oxide also increased. The system offered good results in platelet aggregation inhibition; therefore, it is suitable to be applied to a sensor for health disorder prevention [118]. Mohan et al. [119] obtained, using *Catharanthus* leaf extract and green chemistry, a silver–palladium bimetallic system within 15–30 nm. The material obtained was tested for its photocatalytic effect and for the scavenging activity, which seemed to be improved once the composite was synthesized [119]. Another study for biosensor fabrication was achieved by Zamarchi and Vieira [120], who developed a biosensor based on silver nanoparticles fabricated with pine nut extract as a reducing agent. The nanoparticles were synthesized using green chemistry and the sensor was obtained through an electrochemical approach for paracetamol determination. The biosensor proved to be stable with a good detection limit of 8.50 × 10^−8^ M [120]. Usman et al. [121] synthesized copper and silver nanoparticles using *Citrus sinensis* peels and tested them for antimicrobial, antiviral, and antifungal properties. The method applied led to the fabrication of stable metal nanoparticles with high antimicrobial activity against the different strains tested [121].

## 3. Microfluidic Biosensors: An Overview

Microfluidic systems are miniaturized platforms that enable the precise control and manipulation of small volumes of fluids, typically in the microliter to picoliter range. These systems have gained significant attention in biosensing applications, particularly in the analysis of biological fluids, due to their high sensitivity, reduced reagent consumption, and rapid response times. Microfluidic biosensors have been integrated with metal nanocomposites to enhance detection capabilities, benefiting from the unique optical, electrical, and catalytic properties of nanomaterials [122].

**Figure 4:**
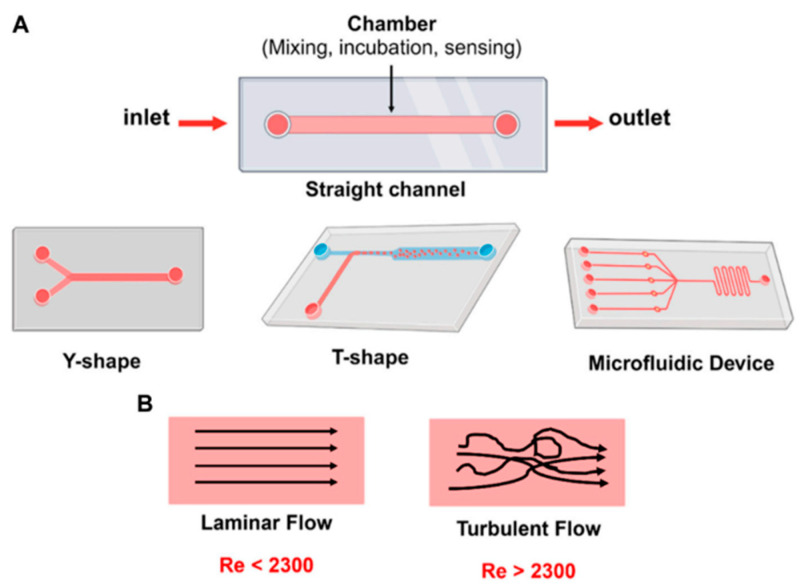
Microfluidic platform designs with various flow geometries: straight channel, Y shaped-channel, and T-shaped channel (**A**) characterized by either laminar or turbulent flow depending on Reynold’s number (Re) (**B**). Reprinted from an open-source article [124].

Microfluidic devices are designed using several fundamental principles governing fluid behavior at small scales. Unlike macroscopic fluid dynamics, microfluidics is dominated by low Reynolds numbers, as can be also observed in Figure 3, where viscous forces predominate over inertial forces, leading to laminar flow. This characteristic enables both a precise fluid control and a predictable mixing behavior, essential for biosensor applications [123]. Microfluidic systems can possess different designs and flow geometries. Various possibilities of microfluidic chip design can be observed in Figure 4.

### 3.1. The Most Used Transport Mechanisms That Govern Microfluidic Systems

Molecular diffusion is the primary mechanism for mixing, in the absence of turbulence, which can be slow, but is predictable and allows for controlled reaction kinetics [125].

Electrokinetic transport consisting in techniques such as electrophoresis and electro-osmosis enable fluid movement using electric fields, useful in lab-on-a-chip (LOC) applications for precise sample handling [126].

Capillary action involving passive fluid flow driven by surface tension, is commonly used in lateral flow assays (LFAs), which are widely utilized in rapid diagnostic tests [127].

Pressure-driven flow with external pumps or syringe-based systems to drive liquid movement is another transport mechanism, crucial in complex biosensing assays requiring controlled fluid delivery [128].

Acoustic-driven transport is a recent advancement using the fact that sound waves can be used to precisely manipulate microfluidic droplets, enabling high-throughput biosensing applications, as revealed by [129].

A magnetically actuated flow is another transport mechanism. The incorporation of magnetic nanoparticles allows for contactless fluid movement, an emerging approach in pathogen detection assays [129].

### 3.2. The Typical Components of Microfluidic Systems Are Briefly Summarized Below

**Microchannels and Chambers**

The microchannel network defines the fluid path and reaction zones. Typically fabricated from materials such as polydimethylsiloxane (PDMS), glass, or thermoplastics, microchannels range from tens to hundreds of micrometers in width [130]. Advances in soft lithography, injection molding, and 3D printing have facilitated complex microfluidic designs for high-throughput analysis, allowing for cost-effective mass production. One example is reference [131], which reports on producing microfluidic channels with designed rectangular cross sectional dimensions of 210 μm actual diameters by a low cost procedure using a low-cost, commercially available 3D printer.

**Sample Introduction and Handling**

Accurate sample loading is crucial for biosensing reliability. Some of the most used techniques for sample loading are briefly presented hereafter.

Micropipette-based manual loading is one of the most used sample introduction techniques in microfluidic biosensors. It provides precise control over small-volume liquid handling, allowing accurate sample delivery into microchannels for diagnostic and analytical applications. This technique is particularly advantageous due to its accessibility, affordability, and compatibility with a wide range of biological samples, including blood, urine, saliva, and other bodily fluids [122]. Micropipette-based manual loading presents several advantages. Unlike automated sample handling systems, micropipette-based loading does not require additional pumping or actuation mechanisms, making it a low-cost solution [127]. Advanced micropipettes can deliver volumes with picoliter precision, essential for quantitative biosensing [126]. Moreover, manual loading allows careful dispensing and reducing reagent and sample consumption, which is particularly useful in cases where samples are scarce, such as rare biomarkers in blood plasma [132]. The micropipette-based manual loading technique is compatible with multiple types of biological samples, making it widely used in POC diagnostics [133]. Despite its advantages, micropipette-based loading presents challenges. Accuracy depends on user experience, introducing potential inconsistencies [133]. Another source of possible error lies in the fact that manual loading is not ideal for large-scale or parallel sample processing applications. Moreover, without proper cleaning, micropipette tips may introduce sample contamination, affecting biosensor reliability [134].

Automated sample handling using micropumps and valves, are currently found in commercial POC devices for pathogen detection [135]. Automated sample handling in microfluidic biosensors relies on micropumps and valves to precisely control fluid flow, improving reproducibility and reducing human error. These components enable continuous, programmable fluid movement, making them ideal for applications requiring high-throughput screening and real-time analysis [122]. There are several types of micropumps in microfluidic systems, such as peristaltic micropumps, which use rotating rollers to create pressure-driven flow [136]. Electrokinetic micropumps operate via electrophoresis or electro-osmosis, ensuring precise control in DNA and protein analysis [137]. Piezoelectric micropumps utilize vibrations from piezoelectric materials to pump fluids, and are used in immunoassays and blood analysis [138]. Capillary and paper-based pumps use passive fluid transport mechanisms found in lateral flow assays [139].

Microfluidic valves play a crucial role in biosensing applications, particularly in the analysis of biological fluids such as blood, saliva, and urine. For example, integrated microvalves have been used in microfluidic immunoassays to control the sequential delivery of reagents and samples, enabling the highly sensitive detection of biomarkers [140]. In another study, a microfluidic system with pneumatic valves was employed for the automated analysis of glucose and lactate in blood samples, demonstrating the potential for POC diagnostics [141].

Microfluidic valves can be broadly categorized into two types: passive and active valves. Passive valves rely on the fluid’s properties and channel geometry to control flow, while active valves use external actuation mechanisms to regulate fluid movement.

Passive valves operate without external energy input and are often designed based on the principles of capillary action, surface tension, or fluid resistance. One common type of passive valve is the check valve, which allows unidirectional flow and prevents backflow. For example, Tesla valves, which utilize a series of asymmetric loops to create a preferential flow direction, have been integrated into microfluidic systems for flow rectification without moving parts [142]. Another example is the hydrophobic valve, which uses surface tension to stop fluid flow at specific points in the channel until a certain pressure threshold is reached [143].

Active valves, on the other hand, require external energy to function and offer greater control over fluid flow. These valves can be actuated using various mechanisms, including pneumatic, piezoelectric, electrostatic, and thermopneumatic methods.

Pneumatically actuated valves are among the most widely used in microfluidics. They typically consist of a flexible membrane (e.g., PDMS) that deforms under air pressure to open or close a fluidic channel. The Quake valve, developed by Unger et al., is a pioneering example of a pneumatically actuated valve that has been widely adopted in microfluidic systems [144]. These valves are highly reliable and can be integrated into complex multiplexed systems.

Piezoelectric materials change shape when an electric field is applied, making them suitable for precise flow control. Piezoelectric valves are often used in applications requiring rapid response times and high-frequency operation. For instance, a piezoelectric actuator integrated into a microfluidic valve demonstrated fast switching times (<1 ms) and precise control over nanoliter volumes [136].

Electrostatic actuation relies on the attraction or repulsion of charged plates to control valve operation. These valves are compact and energy-efficient but typically require high voltages for actuation. An electrostatic microvalve with a low actuation voltage (30 V) was demonstrated for controlling flow in microfluidic channels, showing the potential for portable biosensing applications [143].

Thermopneumatic valves use the expansion of a gas or liquid due to heating to actuate the valve. These valves are advantageous for their simplicity and compatibility with microfabrication techniques. A thermopneumatic valve using a paraffin wax actuator was developed for disposable microfluidic systems, demonstrating reliable operation and low power consumption [145].

### 3.3. Detection and Signal Transduction

Detection and signal transduction are pivotal components of biosensing systems, particularly in the analysis of biological fluids using metal nanocomposites. The integration of metal nanocomposites into biosensing platforms has revolutionized the analysis of biological fluids, enabling rapid, accurate, and portable diagnostics. These systems leverage the unique optical, electrochemical, and mechanical properties of metal nanocomposites to achieve high sensitivity, specificity, and real-time detection capabilities.

Optical detection methods, such as SERS and fluorescence, are widely employed due to the plasmonic properties of metal nanoparticles. For instance, Rissin et al. [146] demonstrated ultrasensitive protein detection using single-molecule arrays (Simoa) with fluorescence-based signal transduction, achieving femtogram-per-milliliter sensitivity. Similarly, Zhang et al. [147] utilized gold nanoparticle-based quantum dots in a microfluidic platform for the multiplexed detection of cancer biomarkers, showcasing the potential of metal nanocomposites in clinical diagnostics.

Electrochemical detection methods are well suited for the real-time monitoring of analytes in biological fluids. Metal nanocomposites, such as gold and platinum nanoparticles, enhance electron transfer and improve sensor performance. Wang et al. [148] developed an electrochemical biosensor for continuous glucose monitoring, highlighting its application in diabetes management. Additionally, Liu et al. [149] demonstrated a microfluidic device incorporating silver nanoparticles for detecting heavy metal ions in water, emphasizing the role of metal nanocomposites in environmental and biological sensing.

Mechanical detection methods, such as cantilever-based sensors, have also benefited from the integration of metal nanocomposites. These materials enhance sensitivity by amplifying surface stress changes during binding events. Burg et al. [150] used microcantilevers functionalized with gold nanoparticles for label-free protein detection, achieving remarkable sensitivity. Furthermore, Lee et al. [151] developed a piezoelectric microcantilever biosensor incorporating metal nanocomposites for the real-time detection of bacterial pathogens, demonstrating the versatility of these materials in diagnostics.

### 3.4. Microfluidic Actuators and Pumps

Microfluidic actuators and pumps are fundamental components in microfluidic biosensors, enabling precise manipulation and transport of fluids at the microscale. These devices are critical for applications such as drug delivery, biological assays, and environmental monitoring. Among the most widely used actuators are piezoelectric, pneumatic, and electrokinetic pumps, each offering unique advantages in terms of flow control, scalability, and integration with microfluidic systems. Additionally, paper-based microfluidic systems have emerged as a low-cost and portable alternative, leveraging capillary action for fluid transport without the need for external pumps. The integration of these actuators and pumps, including paper-based systems, into microfluidic platforms has revolutionized the field, enabling automation, miniaturization, and high precision in fluid handling.

Piezoelectric actuators, which rely on the deformation of piezoelectric materials under an applied voltage, are highly precise and capable of generating high pressures. For example, ref. [136] demonstrated the use of piezoelectric micropumps for high-pressure microfluidic applications, showcasing their utility in complex fluidic networks. Similarly, ref. [152] highlighted the integration of piezoelectric actuators in microfluidic systems for drug delivery, emphasizing their ability to achieve precise dosing.

Pneumatic actuators, which use compressed air or a vacuum to control fluid movement, are another popular choice due to their simplicity and compatibility with soft lithography techniques. Unger et al. [144] developed a pneumatic microfluidic valve and pump system using multilayer soft lithography, enabling the automation of complex fluidic operations in biological assays. This approach has been widely adopted in organ-on-a-chip platforms and high-throughput screening systems.

Electrokinetic pumps, which utilize electric fields to move fluids through electro-osmosis or electrophoresis, are particularly useful for applications requiring low flow rates and minimal mechanical components. Many studies demonstrated the use of electrokinetic pumps for the precise control of nanoliter volumes in microfluidic devices, highlighting their potential for analytical chemistry applications. Additionally, Eijkel and Berg [153] explored the use of electrokinetic pumps in lab-on-a-chip systems for environmental monitoring, showcasing their versatility and efficiency.

Paper-based microfluidics, often referred to as microfluidic paper-based analytical devices (µPADs), have gained attention as a low-cost and portable alternative to traditional microfluidic systems. These devices rely on capillary action to transport fluids through porous paper substrates, eliminating the need for external pumps. Martinez et al. [154] pioneered the development of µPADs, demonstrating their application in POC diagnostics for detecting glucose and protein in biological fluids. Similarly, Yetisen et al. [155] explored the use of paper-based microfluidics for the colorimetric detection of analytes, highlighting their potential for resource-limited settings.

## 4. Integration of Metal Nanocomposites into Microfluidic Biosensors

The integration of metal nanocomposites into microfluidic biosensors has significantly advanced the field of biological fluid analysis. Metal nanocomposites, comprising metal nanoparticles embedded within a matrix, exhibit unique properties such as enhanced electrical conductivity, catalytic activity, and optical characteristics. When incorporated into microfluidic systems, these nanocomposites can substantially improve the sensitivity and specificity of biosensors.

Microfluidic devices, often referred to as LOC systems, manipulate fluids at the microscale, offering advantages like reduced reagent consumption, faster analysis times, and the ability to integrate multiple laboratory functions into a single platform [156]. The fusion of microfluidics with metal nanocomposites enables the development of compact, efficient, and highly sensitive biosensors capable of detecting low concentrations of analytes in complex biological matrices.

Recent studies have demonstrated the potential of this integration. For instance, researchers have developed microfluidic platforms incorporating metallic nanostructures to enhance biosensing capabilities, achieving high sensitivity and specificity in medical diagnostics. These advancements highlight the versatility of combining microfluidic devices with nanomaterials, emphasizing benefits such as real-time detection, portability, and simultaneous analysis of multiple analytes [157].

The convergence of metal nanocomposites and microfluidic technology holds promise for various applications, including POC diagnostics, environmental monitoring, and personalized medicine. By leveraging the unique properties of metal nanocomposites within microfluidic platforms, researchers aim to develop next-generation biosensors that are not only more sensitive but also more robust and versatile, addressing current challenges in biological fluid analysis [158].

A successful integration of noble metal nanocomposites in a microfluidic platform is reported in [159]. The electrode was modified by phosphorene–gold nanocomposites, on which an aptamer specific to Okadaic acid, a widely distributed bio-toxin, was immobilized. The device consisted of channels for sample mixing and incubation. The authors report achieving a detection limit of 8 pM, with a linear range between 10 nM and 250 nM. The authors claim a 10× higher sensitivity as compared to conventional biosensors.

### 4.1. Types of Metal Nanocomposites Used in Microfluidic Biosensors

**Noble Metal-Based Nanocomposites**

Noble metal-based nanocomposites, particularly Au and Ag nanoparticles, have emerged as key materials in the development of advanced microfluidic biosensors due to their unique plasmonic properties. These nanoparticles exhibit LSPR, a phenomenon where incident light induces collective oscillations of conduction electrons, leading to enhanced optical responses. This property makes them highly suitable for optical biosensing applications, including label-free detection, SERS and colorimetric assays.

Gold and silver nanoparticles are widely used in microfluidic biosensors due to their strong LSPR effects, biocompatibility, and ease of functionalization with biomolecules. The functionalization can be obtained through various approaches such as thiol chemistry using oligonucleotide [160], organic molecules such as α-lipoic acids [161], amino acids [162], or polymer coating in order to obtain a core–shell effect for sensitive detection [163]. The LSPR peak of these nanoparticles is highly sensitive to changes in the local refractive index, making them ideal for detecting molecular binding events. For example, ref. [164] demonstrated the use of LSPR-based biosensing, achieving real-time detection of protein interactions with high sensitivity. Similarly, ref. [165] highlighted the application of silver nanoparticles in SERS-based biosensors, where the enhanced electromagnetic fields at the nanoparticle surface enable the detection of trace analytes.

One of the most prominent applications of noble metal nanocomposites is in the development of LSPR-based glucose sensors. The work in [166] reports on a designed microfluidic biosensor incorporating gold nanoparticles functionalized with glucose oxidase for the detection of glucose in biological fluids. The LSPR shift caused by the enzymatic reaction provided a highly sensitive and selective readout, demonstrating the potential of such systems for diabetes management. The work reported in [167] presents a silver nanoparticle-based LSPR sensor for detecting glucose in urine, showcasing the versatility of noble metal nanocomposites in POC diagnostics.

In addition to glucose sensing, noble metal nanocomposites have been extensively used in immunoassays. Jiang et al. [168] developed a microfluidic immunoassay platform using gold nanoparticles for the detection of cardiac biomarkers [168]. The LSPR shift resulting from antigen–antibody interactions enabled quantitative analysis with high sensitivity and specificity. Furthermore, the work reported in [169] demonstrated a silver nanoparticle-based immunoassay for the detection of cancer biomarkers, highlighting the potential of these materials in early disease diagnosis.

The integration of gold and silver nanoparticles into microfluidic biosensors has significantly enhanced the sensitivity, specificity, and portability of these devices. Their plasmonic properties enable real-time, label-free detection of analytes, making them indispensable in the development of next-generation biosensing platforms for healthcare, environmental monitoring, and beyond.

**Magnetic Nanocomposites**

Magnetic nanocomposites, particularly those based on iron oxide (Fe_3_O_4_) nanoparticles, have become indispensable in microfluidic biosensing due to their unique magnetic properties, biocompatibility, and versatility. These nanoparticles enable efficient magnetic separation, target enrichment, and manipulation of analytes within microfluidic systems, making them ideal for applications such as pathogen detection, cell sorting, and immunoassays. Iron oxide nanoparticles exhibit superparamagnetic behavior, meaning they can be magnetized under an external magnetic field but retain no residual magnetism once the field is removed. This property allows for precise control over their movement within microfluidic channels, enabling the separation and enrichment of target analytes from complex biological samples. The authors of [170] demonstrated the use of iron oxide nanoparticles for the magnetic separation of proteins and cells, highlighting their potential for high-efficiency sample preparation in microfluidic systems. Similarly, ref. [171] reports on developing a microfluidic platform incorporating magnetic nanoparticles for the rapid isolation of circulating tumor cells (CTCs) from blood, showcasing their utility in cancer diagnostics.

One of the most impactful applications of magnetic nanocomposites is in the detection of pathogens in whole blood. Traditional methods for pathogen detection often require time-consuming sample preparation steps, but magnetic nanoparticles enable rapid and efficient target enrichment directly within microfluidic devices. Pandey et al. reported, in [172], on developing a microfluidic chip functionalized with iron oxide nanoparticles for the detection of *Escherichia coli*. The magnetic nanoparticles were conjugated with antibodies specific to the pathogen, allowing for their capture and concentration under an external magnetic field. This approach significantly reduced the detection time and improved sensitivity, demonstrating the potential of magnetic nanocomposites for POC diagnostics. Another notable example is the work in [173], who designed a microfluidic system incorporating magnetic nanoparticles for the detection of *Salmonella*. The nanoparticles were used to isolate and concentrate the bacteria, which were then detected using a combination of fluorescent labeling and optical sensing. This integrated approach enabled rapid and accurate pathogen detection, even at low concentrations, highlighting the advantages of magnetic nanocomposites in microfluidic biosensing.

The superparamagnetic properties of Fe_3_O_4_ nanoparticles provide a versatile and efficient alternative to traditional separation methods. An example reported in [174,175] states that superparamagnetic nanoparticles exhibit exceptional potential for drug enrichment and separation due to their high magnetic responsiveness and tunable surface properties.

**Figure 5:**
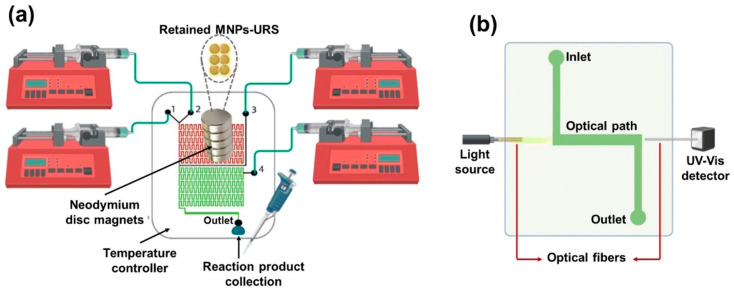
Schematic of a microfluidic device for urea detection using spectrophotometric detection. The first part (**a**) illustrates the flow components for MNPs–URS quantification collected in a micropipette system: (1) inlets with distilled water, (2) the urea in PBS, (3) a hypochlorite solution, and (4) a salicylate solution acting as catalyst. The second part (**b**) represents the microdevice detection system. Reprinted from an open-access article [176].

Magnetic nanoparticles are also used for urea detection from urine and plasma. Chávez-Ramos and Pilar Cañizares-Macías proposed in a recent study the use of a microfluidic system based on magnetic nanoparticles (MNPs) for urea detection (URS) [176]. The schematic representation of the microfluidic platform can be observed in Figure 5.

The microdevice created is based on two zones: the enzymatic area (red) consisting of MNPs-URS where, using a magnet, these nanoparticles are trapped in microchannels, and a detection area (green) where catalysis solutions are added for the reaction to happen. Using this type of device, a shorter analysis time (1 min 40 s vs. 60 min for a classical methodology), less reagents (1.3 μL vs. 100–500 μL for a classical methodology), and considerably less waste volume is achieved [176].

**Metal Oxide Nanocomposites**

Metal oxide nanocomposites, such as zinc oxide (ZnO), titanium dioxide (TiO_2_), and CuO, have gained attention in the field of electrochemical biosensing due to their outstanding electrical, catalytic, and biocompatible properties. These materials are particularly advantageous for enzyme-free biosensors, where their intrinsic catalytic activity enables the direct detection of analytes without the need for biological recognition elements. This simplifies the fabrication process, enhances stability, and reduces costs, making metal oxide nanocomposites ideal for POC diagnostics and environmental monitoring.

ZnO is widely used in electrochemical biosensors due to its high electron mobility, biocompatibility, and ability to form nanostructures with large surface areas. These properties enhance the sensitivity and selectivity of biosensors by facilitating electron transfer and providing abundant active sites for analyte interactions. For example, in [177], it was revealed that the use of ZnO nanorods for the electrochemical detection of glucose achieved high sensitivity and a low detection limit without the need for enzymes. Similarly, ref. [178] described a ZnO-based electrochemical sensor for the detection of hydrogen peroxide, showcasing its potential for environmental and biomedical applications.

TiO_2_ is another promising material for electrochemical sensing due to its excellent chemical stability, photocatalytic activity, and biocompatibility. TiO_2_ nanoparticles have been used to enhance the performance of biosensors by improving electron transfer and providing a stable platform for immobilizing biomolecules. The study in [179] reports on a TiO_2_-based electrochemical sensor for the detection of dopamine, demonstrating its high sensitivity and selectivity in complex biological samples. Additionally, ref. [180] presents TiO_2_ nanocomposites used for the detection of heavy metal ions in water, highlighting their versatility in environmental monitoring.

CuO is known for its high catalytic activity and electrical conductivity, making it a valuable material for enzyme-free biosensors. CuO nanoparticles have been used to detect a wide range of analytes, including glucose, cholesterol, and uric acid. The study in [181] reports on developing a CuO-based electrochemical sensor for the detection of uric acid, achieving excellent sensitivity and a low detection limit. Similarly, ref. [182] demonstrated the use of CuO nanocomposites for the enzyme-free detection of lactate, showcasing their potential for real-time monitoring in clinical settings.

One of the most notable applications of metal oxide nanocomposites is in the development of enzyme-free biosensors for the detection of lactate and uric acid. Lactate is a key biomarker for conditions such as sepsis and hypoxia, while uric acid is associated with gout and kidney disease. Traditional biosensors for these analytes often rely on enzymes, which can be expensive, unstable, and sensitive to environmental conditions. Metal oxide nanocomposites offer a robust alternative by leveraging their intrinsic catalytic properties.

For instance, ref. [183] reports on a ZnO-based enzyme-free biosensor for the detection of lactate in human sweat. The sensor exhibited high sensitivity, a wide linear range, and excellent stability, making it suitable for wearable health monitoring devices. Similarly, ref. [181] presents the successful design of a CuO-based electrochemical sensor for the detection of uric acid in serum samples. The sensor demonstrated high selectivity and a low detection limit, highlighting its potential for POC diagnostics.

MnO_2_ has been extensively studied for non-enzymatic glucose sensing due to its excellent catalytic properties. For instance, a study demonstrated that electrodes modified with MnO_2_ and multi-walled carbon nanotubes exhibited highly sensitive and stable amperometric sensing of glucose at low potentials, making them promising for glucose detection applications [175].

Research has shown that SnO_2_ nanowires can be used for the non-enzymatic detection of hydrogen peroxide (H_2_O_2_). An electrochemical sensor based on graphene-supported SnO_2_ nanoclusters demonstrated effective H_2_O_2_ detection, highlighting the potential of SnO_2_ in biosensing applications [184].

Metal nanocomposites significantly improve electrochemical sensor performance by simultaneously addressing multiple critical parameters. Their enhanced functionality originates from four synergistic mechanisms. First, the incorporation of nanostructured metals dramatically increases the effective surface area of sensing electrodes. This structural advantage provides more active sites for analyte interaction, directly improving sensitivity and signal-to-noise ratios. Studies have demonstrated up to 10-fold sensitivity enhancements in nanocomposite-modified sensors compared to conventional designs [185].

Second, the intrinsic conductivity of noble metal nanoparticles (Au, Ag, Pt) and transition metals (Cu, Ni) create efficient electron transfer pathways. This property reduces charge transfer resistance at the electrode–electrolyte interface, enabling faster response times and more accurate quantification of target species [186]. Recent work shows these materials can decrease response times by 65% while maintaining detection fidelity.

Third, the unique electrocatalytic properties of metal nanocomposites facilitate redox reactions at lower overpotentials. This catalytic enhancement proves particularly valuable for detecting challenging biomolecules like dopamine in complex matrices, where it enables detection limits in the picomolar range [185]. The materials’ selective catalytic activity also improves discrimination against interfering species.

Finally, strategic combination with supporting materials yields additional benefits. Hybrid architectures incorporating graphene derivatives or conductive polymers demonstrate remarkable stability (>6 months) and anti-fouling characteristics [187]. These composites maintain >90% initial sensitivity after 500 measurement cycles in biological fluids.

**Hybrid and Functionalized Metal Nanocomposites**

Hybrid and functionalized metal nanocomposites represent a significant advancement in the field of microfluidic biosensing, offering enhanced catalytic activity, improved stability, and tailored functionality for specific applications. These materials combine the unique properties of different metals or integrate functional groups to achieve superior performance in sensing, catalysis, and target detection. Bimetallic nanoparticles, such as Au–Ag and platinum–palladium (Pt–Pd), are particularly notable for their synergistic effects, while surface functionalization with biomolecules enables highly specific and sensitive detection of target analytes.

Bimetallic nanoparticles leverage the combined properties of two metals to achieve enhanced catalytic activity, improved stability, and tunable optical and electronic properties. For example, Au–Ag nanoparticles exhibit superior plasmonic properties compared to their monometallic counterparts, making them ideal for optical biosensing applications. The work in [188] demonstrated the use of Au–Ag bimetallic nanoparticles for SERS-based detection of biomolecules. Similarly, Pt–Pd nanoparticles have been widely used in electrochemical biosensors due to their exceptional catalytic activity and resistance to poisoning. The study in [189] reports on developing a Pt–Pd-based electrochemical sensor for the detection of hydrogen peroxide, showcasing its high sensitivity and stability in complex biological matrices.

Surface functionalization of metal nanocomposites with biomolecules, such as antibodies, enzymes, or DNA, enables the highly specific and sensitive detection of target analytes. This approach leverages the biorecognition capabilities of biomolecules to achieve selective binding, while the metal nanoparticles provide a robust platform for signal transduction. For instance, ref. [190] reports on functionalizing gold nanoparticles with antibodies for the detection of cardiac troponin I, a biomarker for myocardial infarction. The functionalized nanoparticles were integrated into a microfluidic device, enabling the rapid and accurate detection of the biomarker in serum samples. The study in [191] revealed a DNA-functionalized silver nanoparticle-based biosensor for the detection of microRNAs, demonstrating its potential for early cancer diagnosis.

Another notable example is the work reported in [192] where functionalized platinum nanoparticles with glucose oxidase were used for the development of an enzyme-based electrochemical glucose sensor. The functionalized nanoparticles exhibited high catalytic activity and stability, enabling the detection of glucose in blood samples with high sensitivity and selectivity. This approach highlights the versatility of functionalized metal nanocomposites in biosensing applications.

Another example is presented in [193] which reports using a dual-mode detection strategy for cancer-derived exosomes using graphene field-effect transistors (GFETs). This approach combines two complementary techniques: electrical measurements tracking Dirac point shifts in graphene and Raman spectroscopy identifying optical signatures of cancer-specific biomolecules. By integrating these orthogonal readouts, the reported method enables label-free, high-specificity exosome analysis. The proposed platform is adaptable to multiple cancer types, offering a universal framework for early cancer diagnostics.

The results of the research reported in [194] deal with the immune responses induced by metal-filled single-walled carbon nanotubes (SWCNT) under in vitro, ex vivo, and in vivo settings. Amino-functionalized SWCNTs—either empty, SmCl_3_-filled, or SmCl_3_-filled with Cetuximab—were tested on RAW 264.7 and PBMC cells (1–100 μg/mL, 24 h). Cell viability (unchanged in RAW 264.7) and cytokine release (IL-6/TNFα slightly elevated by one and three) were assessed via flow cytometry and ELISA. PBMC immunophenotyping revealed dose-dependent monocyte/macrophage depletion in vitro, though in vivo mouse studies (150 μg/mouse, days 1/7/13) showed no monocyte reduction. These findings support the further exploration of these conjugates for targeted radiotherapy applications.

Another study [195] presents a flexible 3D carbon nanoweb (3DCNW) aptasensor for the ultrasensitive detection of the oncogenic biomarker PDGF. The sensor was fabricated by electrospinning poly(acrylonitrile) (PAN) nanowebs, followed by Cu-assisted chemical vapor deposition and etching to create textured carbon surfaces. Functionalization with PDGF-B-binding aptamers yielded a biosensor with exceptional performance: 1.78 fM detection limit, high selectivity, reversibility, and stability for PDGF-BB.

**Fabrication Challenges in Metal Nanocomposite-Based Microfluidic Biosensors**

The integration of metal nanocomposites into microfluidic biosensors offers advantages such as enhanced sensitivity, signal amplification, and selective detection. However, challenges related to nanoparticle aggregation during fabrication can severely affect sensor performance by reducing surface reactivity, altering plasmonic properties, and decreasing reproducibility. To mitigate these issues, several strategies have been developed.

Surface functionalization is one method used to prevent aggregation. Biomolecules can effectively stabilize AuNPs within biological environments, while also maintaining some of their biological activities when conjugated to AuNPs. Moreover, functionalizing AuNPs with biomolecules has been shown to greatly enhance their biocompatibility. Such an example is presented in [196], which reports on attaching DNA on AuNPs for the detection of metal ions and small molecules. The authors also present intracellular applications of DNAzyme-functionalized AuNPs.

Another route to prevent aggregation is based on tuned fabrication methods, such as microfluidic-assisted synthesis, which allow for better control over nanoparticle growth and dispersion, preventing aggregation, as reported by [197]. Additionally, layer-by-layer assembly and electrostatic self-assembly techniques enable uniform NP deposition onto biosensor surfaces. Reference [198] describes how the key microfluidic parameters, such as the total flow rate (TFR) and flow rate ratio (FRR) of the aqueous and organic phases, were adjusted to control particle size and improve size distribution. Bulk nanoprecipitation produced larger particles (52–65 nm) with broad size distribution, while microfluidic synthesis yielded smaller (24–43 nm), more uniform nanoparticles.

Another route to prevent aggregation relies on stabilizing ligands and ionic strength control. Metal nanocomposites in biosensors often operate in aqueous solutions, where ionic strength can influence NP stability. By using citrate, PEG, or PVP (polyvinylpyrrolidone) as stabilizing ligands, aggregation can be minimized. Reference [199] reports that monodispersed AgNPs with a core size of around 10 nm coated with PEG, citrate, and PVP were synthesized and characterized; the authors monitored, over a time interval of 21 days, the surface plasmon resonance, size, aggregation, and shape, and found that the charge stabilized particles (citrate) were more unstable than sterically stabilized particles. PVP-stabilized NPs were found to be the most stable, with only small losses in total concentration over 21 days, and no shape, aggregation, or dissolution changes were observed; therefore, the authors recommend them for exposure studies.

NP aggregation can be prevented, while maintaining functionality, by encapsulating metal nanocomposites within porous matrices (hydrogels [200], or polymeric coatings [201,202,203]). This approach is widely used in microfluidic biosensors to improve colloidal stability and long-term performance.

Microfluidic platforms can incorporate continuous-flow systems that dynamically prevent aggregation by maintaining shear forces and controlled mixing. Additionally, in situ functionalization during microfluidic processing ensures nanoparticles remain dispersed throughout the fabrication process [204].

The development of metal nanocomposites for biological fluid analysis requires synthesis methods that are not only effective but also scalable for mass production, particularly in POC diagnostic applications. Among the various techniques available, four approaches have emerged as particularly suitable due to their scalability, cost-effectiveness, and compatibility with biomedical requirements.

Mechanochemical synthesis leverages mechanical energy to drive chemical reactions, enabling the efficient production of metal oxide nanoparticles. For instance, an automated mechanochemical process has been successfully employed to synthesize CuO nanoparticles with high yield and minimal cytotoxicity [205]. This method stands out for its cost efficiency and scalability, making it a viable option for large-scale POC device manufacturing.

Template-assisted synthesis offers precise control over nanoparticle size and morphology by using predefined templates to guide nanostructure formation. This technique ensures consistent quality across production batches, a critical factor for clinical applications where reproducibility is essential [206,207].

Green synthesis provides an environmentally sustainable alternative by utilizing biocompatible solvents and reducing agents, as previously discussed. Beyond its ecological benefits, this approach minimizes nanoparticle toxicity and prevents agglomeration, thereby enhancing the suitability of the resulting nanomaterials for biomedical use [208].

Screen-printing techniques enable the deposition of functional nanomaterials onto diverse substrates, facilitating the mass fabrication of electrochemical sensors. Due to its simplicity, low cost, and scalability, screen-printing has become a widely adopted method for producing POC diagnostic devices [209].

Mechanochemical, template-assisted, and green synthesis methods, along with screen-printing, represent the most scalable and practical approaches for manufacturing metal nanocomposites for POC diagnostics. Their ability to balance production efficiency with performance and biocompatibility makes them indispensable in advancing accessible and reliable diagnostic technologies.

### 4.2. Methods of Integrating Metal Nanocomposites into Microfluidic Devices

The integration of metal nanocomposites into microfluidic devices is a critical step in the development of advanced biosensors. Various techniques have been employed to ensure the precise and stable incorporation of these materials, enabling enhanced sensitivity, selectivity, and functionality, and they are briefly presented below.

**Surface Immobilization Techniques**

Surface immobilization techniques involve the attachment of metal nanocomposites to the walls or surfaces of microfluidic channels, enabling direct interaction with analytes flowing through the device. This approach is particularly advantageous for creating localized sensing regions within the microfluidic network, which can be tailored for specific applications such as immunoassays, enzymatic reactions, or nucleic acid detection.

The functionalization of microfluidic channels with metal nanocomposites typically involves chemical or physical modifications to the channel surface to facilitate the attachment of nanoparticles. Common methods include covalent bonding, electrostatic interactions, and physical adsorption. For example, ref. [210] reports the use of covalent bonding to immobilize gold nanoparticles onto the surface of PDMS microchannels, creating a stable platform for optical biosensing. Similarly, ref. [211] reports on using electrostatic interactions to attach iron oxide nanoparticles to glass microchannels, enabling efficient magnetic separation of target analytes.

Self-assembled monolayers (SAMs) are a widely used surface immobilization technique for functionalizing microfluidic channels with metal nanocomposites. SAMs are organic molecules that spontaneously form ordered layers on surfaces, providing a versatile platform for the attachment of biomolecules such as antibodies, enzymes, or DNA. For instance, the authors of [212] developed a microfluidic immunosensor by functionalizing gold nanoparticles with SAMs for the attachment of antibodies specific to prostate-specific antigens (PSAs). The SAMs provided a stable and oriented immobilization of the antibodies, enabling the highly sensitive and specific detection of PSA in serum samples. Another example is the work reported in [213] which used SAMs to functionalize silver nanoparticles in a microfluidic device for the detection of *Escherichia coli*. The SAMs facilitated the attachment of antibodies specific to the bacteria, enabling their capture and detection using SERS. This approach demonstrated the potential of SAMs for creating highly sensitive and selective biosensors for pathogen detection.

**In Situ Synthesis of Metal Nanocomposites**

In situ synthesis involves the direct formation of metal nanoparticles within the microfluidic channels, enabling precise control over particle size, distribution, and morphology. This method is particularly advantageous for creating highly uniform nanocomposites without the need for external nanoparticle handling or post-processing steps.

Reference [214] reports on in situ synthesis of gold nanoparticles within PDMS microchannels for optical biosensing applications. By flowing a gold precursor and the cross-linking (curing) agent of the polymer, they achieved the formation of gold nanoparticles with tunable plasmonic properties. The synthesized nanoparticles were then used for LSPR-based detection of biomolecules, showcasing the potential of in situ synthesis for creating integrated sensing platforms.

A microfluidic system for the in situ synthesis of iron oxide nanoparticles, which were subsequently used for magnetic separation of target analytes, was reported in [215]. The synthesis process involved the co-precipitation of iron salts within the microchannels, resulting in nanoparticles with controlled size and magnetic properties. This approach eliminated the need for external nanoparticle handling and enabled seamless integration of magnetic separation into the microfluidic device.

**Embedding Metal Nanocomposites within Polymer Matrices**

Embedding metal nanocomposites within polymer matrices [216], such as PDMS or hydrogels, is a versatile approach for integrating these materials into microfluidic devices. This method provides a stable and biocompatible environment for the nanoparticles while maintaining the flexibility and transparency of the polymer.

Such an example is reported in [214]. The authors developed a microfluidic device with PDMS-embedded gold nanoparticles. The proposed integrated device was further used as a sensitive and low-cost LSPR-based biosensor for the detection of polypeptides. This approach enabled the detection of trace amounts of biomolecules with high specificity, demonstrating the potential of polymer-embedded nanocomposites for biosensing applications.

Another example describes the incorporation of silver nanoparticles into a hydrogel matrix within a microfluidic device for controlled drug release [217]. The hydrogel provided a biocompatible environment for the nanoparticles, while the microfluidic system enabled precise control over the release kinetics. This approach was used for the localized delivery of antimicrobial agents, highlighting the potential of polymer-embedded nanocomposites for therapeutic applications.

**Magnetic Trapping of Metal Nanocomposites**

Magnetic trapping is a powerful technique for integrating magnetic nanocomposites, such as iron oxide nanoparticles, into microfluidic devices. This method leverages external magnetic fields to localize and retain nanoparticles within specific regions of the microchannels, enabling targeted sensing or separation.

An example is the work reported in [218] which demonstrated the use of magnetic nanoparticles for trapping *Escherichia coli* from blood. Another example is presented in [219] where iron oxide nanoparticles were coated with PEG and functionalized via avidin-biotin chemistry with an antibody targeting epithelial cell adhesion molecule (EpCAM). When exposed to tumor cells, these targeted nanoparticles were specifically internalized by EpCAM-expressing tumor cells (e.g., BxPC3, a pancreatic cancer cell), while minimal uptake was observed in cells with low EpCAM expression (e.g., CCRF-CEM, a leukemia cell). This approach demonstrated the potential of magnetic trapping for applications in diagnostics and cell therapy.

### 4.3. Enhancement of Sensor Performance Through Nanocomposite Integration

The incorporation of metal nanocomposites into microfluidic biosensors enhances performance through several mechanisms, including signal amplification, improved electron transfer, enhanced biorecognition, and increased surface area. These improvements are critical for applications in biosensing, where high sensitivity and specificity are required.

**Signal Amplification**

Metal nanocomposites, particularly those with plasmonic properties such as gold and silver nanoparticles, are widely used for signal amplification in optical biosensors. The LSPR effect of these nanoparticles enhances the electromagnetic field at their surface, leading to increased sensitivity in detection methods such as SERS and fluorescence.

Such an example is [219], which reports on developing a PCR-free SERS-based DNA detection method using a dual-platform system of graphene oxide (GO) and AuNPs. This system utilized GO-AuNPs functionalized with capture probe 1 and AuNPs modified with capture probe 2 and a Raman dye (Cy3), enabling hybridization with target DNA sequences. The formation of localized “hot spots” at nanostructure junctions significantly amplified SERS signals, achieving a detection limit as low as 10 fM. This level of sensitivity is critical for early disease diagnosis and highlights the role of metal nanocomposites in signal amplification.

**Improved Electron Transfer**

In electrochemical biosensors, metal nanocomposites such as platinum, gold, and iron oxide nanoparticles enhance electron transfer between the analyte and the electrode, leading to improved sensitivity and lower detection limits. The high surface area and catalytic activity of these nanoparticles facilitate faster and more efficient redox reactions.

Reference [220] reports on growing carbon nanotubes (CNTs) in situ by microwave plasma chemical vapor deposition (MPCVD) followed by the electrochemical addition of Pt nanospheres to form a CNT/Pt nanosphere composite biosensor. The sensor achieved a glucose detection limit and response time of 380 nM and a response time of 8 s.

**Enhanced Biorecognition**

Metal nanocomposites can be functionalized with biomolecules such as antibodies, enzymes, or DNA to enhance the specificity and sensitivity of biosensors. The high surface area of nanoparticles allows for the immobilization of a large number of biorecognition elements, increasing the likelihood of target binding.

An example of enhanced biorecognition is presented in [221] where the authors developed and optimized an advanced sandwich immunogold assay for the highly sensitive detection of cardiac troponin I (cTnI) in human serum. This detection method employs AuNP labeling followed by silver staining, allowing signal amplification through AuNP-induced chemical silver deposition. Compared to conventional direct immunogold detection methods, the described method enhanced method improves cTnI detection sensitivity by two orders of magnitude.

**Increased Surface Area**

The high surface-to-volume ratio of metal nanocomposites provides a larger active area for analyte binding and reaction, leading to improved sensor performance. This is particularly important in microfluidic systems, where the small channel dimensions limit the available surface area.

High aspect ratio zinc oxide nanorods (ZNRs) were grown vertically on electrode surface via a simple one-step low temperature solution route, as reported in [222]. Such electrodes revealed enhanced performance with attractive analytical responses for uric acid (UA), such as a high sensitivity of 239.67 μA cm^−2^ mM^−1^ in a wide-linear range (0.01–4.56 mM), rapid response time (~3 s), low detection limit (5 nM), and low value of apparent Michaelis–Menten constant (Kmapp, 0.025 mM). These results were attributed to the high aspect ratio of vertically grown ZNRs which provide high surface area leading to enhanced enzyme immobilization, high electrocatalytic activity, and direct electron transfer during electrochemical detection of UA.

**Stability and Reusability**

Metal nanocomposites also improve the stability and reusability of biosensors by providing a robust platform for immobilizing biorecognition elements. The chemical and mechanical stability of these materials ensure long-term performance, even in harsh environments.

The effect of Fe_3_O_4_ NPs on urease immobilization using different concentrations of Fe_3_O_4_ NPs was studied in [223]. The authors found that the morphological features for alginate/magnetite Fe_3_O_4_ NPs before and after immobilization were studied. The reusability, half-life, enzymatic kinetics, and storage stability of the enzyme were all enhanced. The authors report that the immobilized urease was reused 20 times and a recovery of 59% of the initial activity.

The integration of metal nanocomposites into microfluidic biosensors has significantly enhanced sensor performance, enabling the detection of analytes at ultra-low concentrations with high specificity and stability. These advancements are supporting the development of next-generation biosensors for applications in healthcare, environmental monitoring, and beyond.

**Practical Challenges and Solutions in Integrating Nanocomposites into Microfluidic Platforms**

The development of metal nanoparticle-based biosensors faces major challenges due to non-specific adsorption (NSA) and nanoparticle leaching, which can negatively impact sensitivity, specificity, and reproducibility. To address these issues, researchers have investigated different strategies, including both passive and active approaches [224].

Passive approaches primarily focus on surface modifications to prevent undesired adsorption. One possibility consists of using materials such as PEG and oligo(ethylene glycol) (OEG) to form hydrophilic, non-ionic layers that resist protein adsorption. However, these coatings may degrade under oxidative conditions, limiting their long-term effectiveness [224]. SAMs are another route which consist of employing alkanethiol SAMs to create dense monolayers with functional groups for bioreceptor attachment. While effective, challenges include imperfections in monolayer formation and compatibility limited to specific substrates like gold and silver [225]. Zwitterionic polymers possess both positive and negative charges, forming strong hydration layers that repel nonspecific interactions. Despite their effectiveness, the synthesis and application of zwitterionic coatings can be complex and may not be suitable for all biosensor platforms [226,227].

Active strategies involve dynamic processes to remove or reduce NSA. Applying external forces, such as electric potentials, has been shown to effectively reduce nonspecific interactions. For instance, research demonstrated that applying an external potential minimized site-preferred nonspecific interactions in single-nanoparticle biosensors [228]. Physical surface modification generally preserves the original chemical composition of the surface. One of the simplest and most widely used strategies to prevent NSA involves coating surfaces with blocker proteins. Common examples include serum albumins (e.g., bovine serum albumin, BSA) [229,230], casein [193,230], and other milk proteins [227], which are frequently employed as blocking agents in techniques such as enzyme-linked immunosorbent assay (ELISA).

Addressing nanoparticle leaching involves ensuring robust attachment of nanoparticles to sensor surfaces. Techniques such as strong covalent bonding and cross-linking agents are employed to enhance nanoparticle stability and prevent detachment during sensor operation. Combining these methods, careful surface modification to prevent NSA, and robust nanoparticle attachment techniques, the performance and reliability of metal nanoparticle-based biosensors is enhanced.

## 5. Detection of Emerging Biomarkers in Biological Fluids

### 5.1. Glucose

Biosensors are intensively studied for glucose determination since diabetes has become a challenging health problem worldwide [231,232]. Besides cancer and antimicrobial resistance, diabetes affects people of different ages, from children to adults. In 2019, 1.5 million deaths were caused by diabetes, with 48% cases occurring at people under 70 years [233]. It is estimated that until 2030, 552 million people will be affected by diabetes [234]; therefore, the need for non-invasive solutions is extremely important.

Glucose is found in the human body, and is used mainly to provide energy for cell metabolism. Besides blood, glucose is found in many other fluids such as saliva, tears, interstitial fluids, or urine [235]. The WHO introduced the standard values for glucose detection from blood as between 70 mg/dL (3.9 mmol/L) and 100 mg/dL (5.6 mmol/L) for health patients [236]. A higher range of 100 to 125 mg/dL (5.6 to 6.9 mmol/L) is an alarm signal to monitor glycemia levels [237].

Most papers classified glucose sensing techniques into invasive, minimally invasive, and non-invasive techniques [238,239]. Invasive techniques usually involve glucose measurement from blood in hospitals or using household glucometers. The results from blood analysis are accurate and precise; however, the use of needles, a visit to the hospital, or the risk of possible infections once the blood is exposed are uncomfortable for the patient. Also, the storage of the samples is important since improper storage would interfere with the accuracy of results [240]. Non-invasive techniques consist of optical methods, microwave methods, or electrochemical methods. The main advantages of such techniques are the detection of glucose without causing damage to the tissues and the lower time of investigation [240].

**Figure 6:**
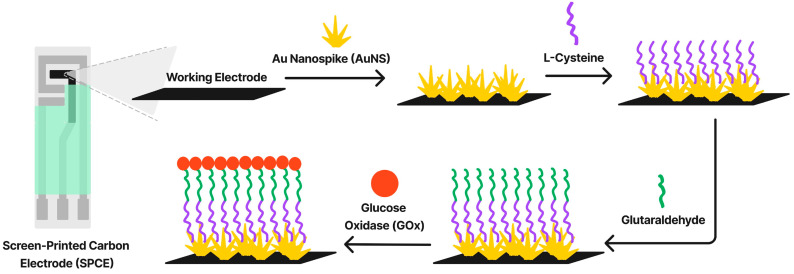
Fabrication scheme for enzymatic glucose biosensor based on gold nanospike-modified screen-printed carbon electrodes. Reprinted from an open-access article [243].

Biosensors are usually considered non-invasive methods since they are ready to use, have high selectivity, and low limits of detection. They offer comfort and they can be applied to different body fluids. Regarding glucose, the major biosensors are based on electrochemical approaches since this type of fabrication method assures the highest sensitivity, reproducibility, low-cost, and easy maintenance [241]. The detection of glucose using an electrochemical biosensor is usually based on the interaction of glucose with different enzymes: hexokinase, glucose oxidase, or glucose-1-dehydrogenase [241]. Glucose oxidase is the standard method for this detection since the enzyme is cost-effective, can be easily obtained, has high sensitivity, and can be subject to chemical, pH, or ionic modifications in experiments [241,242]. The addition of nanomaterials increases the novelty of the system, while assuring different properties at the same time. Many studies were conducted for glucose determinations with the desire to obtain non-invasive devices able to easily detect the levels of glucose from the body. Majidah et al. [243] deposited gold nanospikes into a biosensor using the self-assembled monolayer method, as described in Figure 6. The gold nanospikes were deposited into a screen-printed carbon electrode and the group studied the potential of immobilizing glucose oxidase using L-cysteine. The results proved a successful immobilization of the enzyme and a great limit of detection of 0.2–15 mM in neutral pH [243].

### 5.2. Salivary Biosensors

Saliva is a complex body fluid which can be used to determine various medical issues, starting from a simple infection to an extremely complicated condition such as oral cancer. Saliva is preferable for point-of-case tests since it can be easily collected in sufficient quantities with low risk and non-invasive techniques. The test is usually performed in minutes, a very short time, and represents a fast disease tracking procedure without any risks.

**Figure 7:**
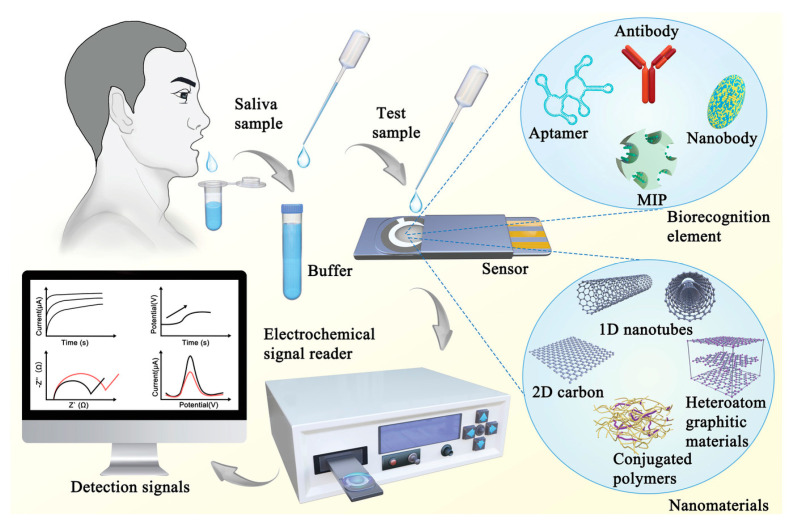
Electrochemical detection of protein biomarkers based on nanomaterials. Reprinted from an open-source article [244].

Proteins are found on the surfaces of viruses or bacteria involved in various diseases. Proteins are usually used as biomarkers for detection and diagnosis [244]. Saliva biosensors and devices were rapidly developed in huge numbers and forms once the global pandemic caused by SARS-CoV-2 was announced. The tests were based on the antibody–antigen interaction, since antibodies for coronavirus were placed inside the tests. The results were on point; therefore, in the time waiting for the PCR results, infected people could protect each other, a fact which decreased infection transmissions. In salivary biosensors, ELISA and Western blot techniques are used to detect, by a colorimetric method, the presence of the antigen. The addition of the enzyme leads to a colorimetric change in the reaction, which is driven by a strong interaction between the antibody in the test and the surrounding solution. However, these techniques require time, so biosensors are an easy alternative for protein detection. In the detection of salivary biomarkers with biosensors, the most studied method is the electrochemical one, as described in Figure 7. Nanomaterials are used together with the antibody to increase the properties of the biosensor and improve its detection.

The field of biosensors using nanomaterials is quite new; therefore, the number of papers in the literature is limited. Samavati et al. [245] reported the fabrication of a miniaturized Au/fiber Bragg grating sensor decorated with graphene oxide to detect SARS-CoV-2 from saliva using an optical spectrum analyzer. The wavelengths and amplitudes were recorded before and after the exposure to saliva, and the results showed a modulation of the response once the virus was detected by the system [245]. Wityk et al. [246] developed a reusable electrochemical biosensor which can detect SARS-CoV-2 RNA from unfiltered saliva. The novelty of the system consists in using the impedance spectroscopy as a detection method, with an optimization in the cuvette design which allows the detection of RNA at very small concentrations [246]. Tlili et al. [247] designed a carbon nanotube chemoresistive immunosensor for the detection of α-amylase without labeling. This amylase is an indicator of the nervous system; therefore, it is associated with stress. The single-walled carbon nanotube biosensor was achieved by a lithographic bridge decorated with gold microelectrodes functionalized with antibodies. The obtained immunosensor presents a limit detection of 6 μg mL^−1^ in PBS and 7.8 μg mL^−1^ in synthetic saliva [247]. Another application for the biosensors in salivary diagnosis is related to the determination of oral cancer. In a review paper, Sethuraman et al. [248], various examples of nanomaterials applied for cancer determinations are mentioned.

## 6. Recent Advances and Applications

### 6.1. Biosensors in Health Care Issues

Biosensors have facilitated the detection of various medical issues. The shorter time of detection and the ability to obtain information in time for treatment are the main advantages of the biosensor field. Rakhshani et al. [249] designed a nanosensor for the detection of high concentrations of hemoglobin able to prevent anemia as disease. Such a device proved to be a real solution in the prevention of anemia, especially in the identification of the different anemia stages, from healthy patients to patients with severe illnesses. The main advantage of the device fabricated by the group is the quick tracking of their hemoglobin levels without major effort and without sacrificing comfort [249]. Another type of plasmonic nanosensor based on metal nanoparticles and graphene layers was achieved by Chahkoutahi et al. [250] for hemoglobin concentration determinations. The graphene–plasmonic-based nano structure sensor was designed using the refractive index approach which proved to have the most accurate results in recent times [251]. The results of the study showed that by increasing the graphene and Au layers, the absorption of the sensor also increased. Therefore, the structures proposed in the study were considered great candidates for the detection of various biomolecules from tissues [250].

### 6.2. Detection of Biological Molecules and Metals in Water

The pollution of water increases every day due to external factors and environmental issues. Water quality can be altered by inorganic pollutants such as chlorine, sulphate, pH, or nitrogen [252]. The main polluting agents are chemicals and biological molecules/toxins, which are a real threat to the ecosystems and to human health. Bacteria, viruses, or protozoa can lead to chronic health problems if the water supply is contaminated. Cholera, typhoid, or shigella can cause people to become sick if the water is not decontaminated [252]. Nowadays, the classical detection methods are chromatography and spectrometry; however, the need for specialized personnel, expensive reagents, and complicated protocol encourages the use of biosensors for precise detection [253]. Besides the detection of chemicals and biological toxins, biosensors are also used with nucleic acids, known as aptamers, allowing for detection based on the classical antibody/antigen relationship. Aptamers are known as single-stranded nucleic acids, RNA, or single-stranded DNA which can attach with high affinity to small molecules, ions, proteins, and cells based on a specific interaction [254]. The biosensor consists of a known matrix which will interact with the specific antigen from the water sample, for example, with various bacteria strains. Aptamers are used in various studies for bacteria detection, as reported by Zhang et al. [255]. Biosensors are also used in water purification and heavy metals removal from aqueous solutions due to the catalytic properties that they offer. A recent paper published by Mirghani M. [256] proposed a nanocomposite based on molybdenum-doped titanium dioxide (Mo-doped TiO_2_) nanoparticles to remove Cr (VI) from aqueous solutions. The results proved that the photocatalytic effect of the system, fabricated using a sol–gel method, increases while a nanocomposite is obtained. The removal percentage for TiO_2_ was 41%, while for Mo-doped TiO_2_, it was confirmed at 90% [256]. Farhan et al. [257] described in a review article the use of metal nanocomposites based on carbon materials for toxic metal removal based on photocatalytic oxidation [257]. Velusamy et al. also reported the use of biosensors for water pollution due to their sensitivity and selectivity. The article also mentioned the detection limit for heavy metals in recent years, which is assumed at 65.36 ng/mL [258]. Yang et al. [259] developed a nanocomposite surface-functionalized glassy carbon electrode doped with platinum nanoparticles to immobilize acetylcholinesterase for pesticide detection. The biosensor presents a detection limit of 5 × 10^−14^ M for parathion and 5 × 10^−13^ M for carbofuran, showing good sensitivity and stability for pesticide detection [259].

### 6.3. Detection of Food Pathogens Using Biosensors

Foodborne pathogens are a real public health issue which affect humans worldwide. Raw meat, milk, or vegetables are usually the source of contamination and once they are not carefully handled, the entire food supply chain can be affected. The standard method of detecting bacteria from food samples is by inoculating an agar plate, a process which usually requires several days and a very sterile work area, or by the PCR technique [260]. Biosensors will facilitate bacteria detection in a shorter time, compared to classical techniques, while increasing the sensitivity of the test [261]. Zhang et al. [262] proposed an aptamer biosensor based on colorimetric AuNPs for the detection of *Salmonella enteritidis*. In the study, two synthesized aptamers with high affinities were further integrated into AuNPs. Once the aptamers were attached to the nanoparticles, the aggregation was inhibited. The results showed that in presence of bacteria, the aptamer dissociates from the system created and nanoparticle aggregation occurs. The obtained biosensor had a good limit detection of about 10^4^–10^5^ CFU/mL; therefore, the study was considered a successful one [262]. Another study conducted by Wu et al. [263] created an antibody-aptamer-based sensor using peroxidase–gold nanoparticles for the detection of *Salmonella enterica* in milk samples. Once the antibody was successfully attached to the nanoprobes used to amplify the binding of bacteria to the nanoparticles, the assay could have a detection up to 10^3^ CFU mL^−1^ in less than 3 h [263]. *Escherichia coli* is another important bacterium known as a food pathogen, especially due to its rapid response once it targets the body. Kalele et al. [264] fabricated a biosensor based on rabbit immunoglobulin-G antibody-conjugated silver nanoshells to identify *Escherichia coli* at a higher sensitivity. Based on the presence of *Escherichia coli* cells, an alteration in the band shift of nanoparticles was identified using surface plasmon resonance techniques, and the detection was considered an optimal one [264]. In a recent paper, Soysald et al. [265] developed a paper-based biosensor with magnetic core–shell Fe_2_O_3_@CdSe/ZnS quantum dots (MQDs) for faster detection with high sensitivity, with the potential to be transformed in a point-of-case device. The biosensor was obtained using electrochemical impedance spectroscopy and the detection was achieved in just 30 min with a limit detection of 2.7 × 10^2^ CFU/mL [265].

### 6.4. Translation of Research into Commercial Products

In recent years, metal nanocomposite-based microfluidic biosensors have made impressive strides, becoming more sensitive, precise, and portable. These advancements are not just expanding the frontiers of research, they are also making their way into real world applications, solving challenges in healthcare, environmental monitoring, food safety, and more. The journey from laboratory research to commercially viable products involves overcoming several challenges, including scalability, cost-effectiveness, regulatory approval, and having a user-friendly design. There are many devices that are currently used for continuous glucose monitoring for diabetic patients; as portable devices for the detection of infectious diseases such as HIV, tuberculosis, and COVID-19; for environmental monitoring designed to detect pollutants, heavy metals, and pathogens in water and air; and for food safety testing, enabling the rapid detection of pathogens, toxins, and allergens in food products. Other devices are used in clinical laboratories for the early detection of prostate cancer. There are devices that provide real-time monitoring of electrolyte levels during physical activity and others that offer microfluidic drug delivery systems, enabling the precise and controlled release of therapeutic agents.

Industrial process monitoring is another area where the microfluidic biosensors are also being used, providing a cost-effective and reliable alternative to traditional laboratory methods, making them ideal for use in chemical and pharmaceutical industries.

It is difficult, at this point in time, to associate a particular technology and a procedure with any of the scientific reports mentioned in this paper, because the technologies are protected by patents and the device producers do not precisely indicate the details of the processes and materials used; therefore, this subsection will not be detailed further.

Despite the successful translation of some of the several metal nanocomposite-based microfluidic biosensors into commercial products, several challenges remain. These include the need for further improvements in scalability, cost-effectiveness, and regulatory approval. Additionally, the development of user-friendly designs and the integration of wireless connectivity and data analytics are critical for the widespread adoption of these devices.

Future research should focus on addressing these challenges and exploring new applications for metal nanocomposite-based microfluidic biosensors. For example, the integration of artificial intelligence (AI) and machine learning (ML) algorithms could enable real-time data analysis and decision-making, further enhancing the utility of these devices.

## 7. Challenges and Future Perspectives

The development of metal nanocomposite-based biosensors has made significant advances in recent years, offering unprecedented sensitivity, specificity, and portability. However, several technical and practical challenges must be addressed to fully realize their potential and facilitate widespread adoption. We discuss below the key challenges in developing these biosensors and explore potential solutions to overcome them.

### 7.1. Technical and Practical Challenges in Developing Metal Nanocomposite Biosensors

The integration of metal nanocomposites into microfluidic biosensors presents a unique set of challenges that span material science, engineering, and regulatory compliance. These challenges must be addressed to ensure the reliability, scalability, and commercial viability of these devices.

**Fabrication Complexity and Scalability**

The fabrication of metal nanocomposite-based microfluidic biosensors often involves complex processes, such as nanoparticle synthesis, surface functionalization, and precise integration into microfluidic channels. These processes can be time-consuming, costly, and difficult to scale up for mass production.

Gold nanoparticles were synthesized in a PDMS microfluidic chip using an in situ method, leveraging the reductive properties of the PDMS cross-linking agent, resulting in improved size uniformity (8% variation vs. 67% in macro-scale synthesis) [214]. The integrated device demonstrated high sensitivity (74 nm/RIU) and a low detection limit (3.7 ng/mL) for bovine growth hormone, highlighting its potential for clinical protein detection. Nevertheless, the reaction in microchannels is found to be slower than at the macro-scale, and scaling up is not straightforward. Advances in automated fabrication techniques, such as roll-to-roll printing and microcontact printing, could simplify the manufacturing process and improve scalability.

Nanocomposite fabrication faces specific challenges, like batch-to-batch variability in nanoparticle size. Such limitations may be resolved via automated synthesis platforms. One such alternative is related to CRISPR, which is short for “clustered regularly interspaced short palindromic repeats”. This is a technology that is used to selectively modify the DNA of living organisms. CRISPR was adapted for use in the laboratory from naturally occurring genome editing systems found in bacteria. Such an example is reported in [266] about metal–organic frameworks (MOFs), which are highly porous materials with tunable structures, widely used in catalysis, and sensing. In biosensing, MOFs are combined with CRISPR–Cas systems (CRISPR–Cas being an adaptive immune system existing in most bacteria and archaea, preventing them from being infected by phages, viruses, and other foreign genetic elements [267]) to detect biomolecules (DNA, RNA, proteins) with high sensitivity and specificity. These MOF–CRISPR biosensors leverage CRISPR’s precise targeting and MOFs’ ability to protect, deliver, and enhance signal transduction, offering the advantages of rapid detection, portability, and cost-effectiveness compared to traditional methods.

Four-dimensional printing (4DP) enables 3D-printed objects to undergo programmed shape transformations when triggered by external stimuli. An example of the successful use of 4D printing is reported in [268], where the authors developed a wearable smart sensor by FDM 4D-printing thermoplastic polyurethane (TPU) onto fabric. The stimuli-responsive TPU enabled the monitoring of vital signs such as heart rate, blood pressure, and body temperature, thus demonstrating the potential for real-time health monitoring and early disease detection using smart wearable sensors.

**Stability and Long-Term Performance**

Iron oxide nanoparticles are widely used in microfluidic biosensors due to their magnetic properties, which facilitate the manipulation and detection of biological targets, as described earlier in this paper. However, over time, these nanoparticles can undergo oxidation, leading to a reduction in their magnetic properties and, consequently, a decrease in detection efficiency.

The authors of [269] investigated the fabrication of iron oxide nanoparticles and their magnetic properties. The researchers found that the oxidation of iron oxide nanoparticles can alter their magnetic behavior, which is critical for their performance in biosensing applications.

Another study explored the biologically dynamic degradation of iron oxide nanoparticles using a continuous flow system. The findings revealed that factors such as pH, the presence of proteins, redox species, and chelating agents can influence the degradation rate of iron oxide nanoparticles. This degradation affects their morphology, aggregation state, and magnetic properties, which are essential for their efficacy in applications like magnetic resonance imaging and biosensing [270].

Additionally, research on microfluidic biosensing systems utilizing magnetic nanoparticles has highlighted the importance of maintaining the stability of iron oxide nanoparticles to ensure consistent magnetic properties and detection efficiency [271]. The study emphasizes that the oxidation and degradation of iron oxide nanoparticles can compromise the performance of microfluidic biosensors.

**Biocompatibility and Toxicity**

The incorporation of metal nanoparticles, such as silver and copper, in biosensors has raised concerns regarding their biocompatibility and potential toxicity, especially for in vivo applications. These nanoparticles can release toxic ions over time, which may adversely affect biological tissues.

Reference [272] reveals the result of the investigation of the effects of silver and copper nanoparticles on both bacterial and mammalian cells. The findings indicated that while these nanoparticles exhibited varying levels of toxicity to bacterial cells, they generally showed low toxicity to mammalian cells, except for polyvinylpyrrolidone-coated copper nanoparticles (PVP-CuNPs). The study also highlighted that the release of metal ions from these nanoparticles could induce the generation of reactive oxygen species (ROS), leading to oxidative stress and potential cellular damage.

Another study examined the ion-release kinetics and ecotoxicity effects of silver nanoparticles [273]. The research demonstrated that silver nanoparticles could release silver ions (Ag^+^) into their surroundings, contributing to their overall toxicity. The study emphasized that the dissolved silver ions played a significant role in the observed toxic effects, underscoring the importance of considering ion release when evaluating nanoparticle toxicity.

**Signal Interference and Noise**

The performance of metal nanocomposite biosensors can be significantly compromised when operating in complex biological fluids like blood or urine. These fluids introduce signal interference and noise, diminishing the accuracy and reliability of the sensors. A primary challenge is NSA, where unintended proteins or biomolecules adhere to the sensor surface, leading to elevated background signals and potential false positives.

In impedance biosensing, the presence of background non-specific binding dictates detection limits. A study utilizing a differential impedance biosensor with poly-L-lysine-polyethylene glycol-biotin-coated gold electrodes detected streptavidin in the presence of 0.1% fetal calf serum. The lowest detectable streptavidin concentration was an order of magnitude higher compared to measurements without background proteins, highlighting the impact of non-specific interactions on sensitivity [274].

To mitigate NSA, active removal methods have been developed. These methods generate surface shear forces stronger than the adhesive forces of weakly bound NSA molecules, effectively removing them without displacing specifically bound targets. Techniques such as alternating current electrohydrodynamics (ac-EHD) induced nanoshearing and hypersonic resonance have been employed to enhance sensor performance by reducing NSA [224].

**Cost and Accessibility**

The high cost of materials and fabrication processes poses a significant challenge to the accessibility of metal nanocomposite-based biosensors, especially in resource-limited settings. Addressing this issue requires innovative approaches to reduce costs while maintaining sensor performance.

One promising strategy involves the use of plant-based synthesis methods for metal nanoparticles. These green synthesis techniques utilize plant extracts as reducing agents, offering a cost-effective and scalable alternative to traditional chemical synthesis methods. Additionally, these nanoparticles can serve dual functions in biosensors, acting as both transducers and recognition elements, thereby simplifying sensor design and reducing costs [275]. This approach has been successfully applied in the development of optical and electrochemical biosensors for applications in healthcare diagnostics and environmental monitoring.

Another cost-reduction strategy is the adoption of inkjet printing technology for the fabrication of biosensors. Inkjet printing is a non-contact, additive manufacturing technique that allows for the precise deposition of nanoparticle-based inks onto substrates. This method is advantageous due to its speed, low material consumption, and compatibility with large-scale production [276]. For instance, inkjet printing has been used to deposit silver nanoparticle dispersions to create conductive tracks for biosensor applications. This technique not only reduces material waste but also lowers fabrication costs, making it suitable for producing affordable biosensors.

**Regulatory and Standardization Challenges**

The commercialization of biosensors requires compliance with stringent regulatory standards, which can be a lengthy and costly process. Additionally, the lack of standardized protocols for testing and validation can hinder the development and adoption of these devices.

**Integration with Data Analytics and Connectivity**

Integrating metal nanocomposite-based biosensors with data analytics platforms and wireless connectivity for real-time monitoring and decision-making introduces technical challenges, notably concerning power consumption and data security.

Wireless biosensors require continuous power for data transmission and sensor operation, which can be particularly demanding in portable or implantable devices. Strategies to address this issue include developing energy-efficient communication protocols, utilizing low-power electronics, and incorporating energy harvesting technologies to extend device operational life.

The transmission of sensitive health data over wireless networks raises significant security and privacy concerns. Implementing robust security measures, such as multi-factor authentication (MFA), biometric verification, and cryptographic keys, is essential to protect against unauthorized access and ensure data integrity [277]. However, these measures must be balanced with user-friendliness to encourage widespread adoption.

### 7.2. Future Directions

To facilitate the large-scale production and commercialization of metal nanocomposite-based biosensors, several key areas require further research and innovation.

Scalable and cost-effective nanomaterial synthesis can be achieved by developing green synthesis methods using biological agents (e.g., plant extracts, bacteria) to reduce costs and environmental impact. Moreover, optimizing continuous-flow and microfluidic-assisted synthesis for high-yield and uniform nanoparticle production is another direction to achieve the above-mentioned goal.

Standardization and quality control requires establishing precise characterization techniques to ensure uniformity in nanoparticle size, shape, and surface properties, which can be accomplished by implementing AI-driven process monitoring and automated quality assurance systems to enhance reproducibility.

Advanced manufacturing techniques are necessary to assure reproducibility and the parameters of the metal nanocomposites. Such high-throughput methods might be roll-to-roll printing, inkjet printing, and 3D nanoprinting for efficient biosensor fabrication. Integrating flexible electronics with microfabrication technologies to improve sensor performance and scalability will be another step to achieve this goal.

A significant advancement in designing and producing wearable devices relies on designing ultra-low-power communication protocols and energy-harvesting systems to enable real-time, long-term monitoring together with the integration of digital and wireless technologies, and with enhancing biosensor compatibility with IoT platforms for improved data processing, transmission, and security.

Improved biocompatibility calls for conducting in-depth toxicity studies to ensure safe use in medical and environmental applications. This, combined with standardized testing protocols and aligning with regulatory frameworks (e.g., FDA, ISO), will get the metal nano-composited biosensors closer to clinical adoption.

Another direction that should be investigated refers to sustainability and end-of-life management. This involves investigating biodegradable materials and eco-friendly alternatives to minimize environmental impact and developing efficient recycling or disposal strategies for used biosensors to support a circular economy.

By addressing these research gaps, the transition from laboratory-scale development to industrial production can be accelerated, ensuring that metal nanocomposite biosensors are both accessible and sustainable for widespread use.

### 7.3. Future Perspectives of Metal Nanocomposite-Based Biosensors for Biological Fluid Analysis

The future of metal nanocomposite-based biosensors for biological fluid analysis is poised for significant advancements, driven by continuous innovation in nanomaterials, fabrication techniques, and analytical methodologies. As the demand for fast, accurate, and portable diagnostic tools increases, metal nanocomposites will play a crucial role in shaping the next generation of biosensing technologies.

One of the most promising directions is the integration of biosensors with wearable and implantable devices, enabling real-time, continuous monitoring of biomarkers in biological fluids such as sweat, saliva, blood, and urine. This advancement is particularly relevant for chronic disease management, including diabetes, cardiovascular conditions, and neurodegenerative disorders, where continuous monitoring can facilitate early intervention and personalized treatment plans.

Moreover, the combination of metal nanocomposites with AI and ML is expected to revolutionize biosensing by enhancing data processing, improving detection accuracy, and enabling predictive diagnostics. AI-driven biosensors can analyze complex biological data in real time, offering higher precision in disease detection and reducing false positives or negatives.

Another crucial aspect of future developments is the miniaturization and affordability of biosensors, making them more accessible for POC diagnostics, particularly in resource-limited settings. Advances in microfluidics, flexible electronics, and nanomanufacturing will contribute to the widespread deployment of biosensors in clinical and home-based healthcare applications. Additionally, the shift towards non-invasive and minimally invasive sampling techniques, such as sweat- or breath-based biosensing, will further enhance patient compliance and convenience.

Despite these promising advancements, several challenges must be addressed before widespread adoption. Regulatory approvals, clinical validation, and large-scale reproducibility remain significant hurdles. Ensuring the biocompatibility, stability, and long-term performance of metal nanocomposite-based biosensors is essential for their transition from laboratory research to commercial applications. Furthermore, sustainability concerns related to the synthesis and disposal of nanomaterials must be tackled to develop environmentally friendly alternatives.

In conclusion, metal nanocomposite-based biosensors are set to redefine biological fluid analysis, offering unparalleled sensitivity, selectivity, and real-time monitoring capabilities. With interdisciplinary collaborations between nanotechnology, biomedical engineering, and computational sciences, the future holds immense potential for breakthroughs in precision medicine, decentralized healthcare, and early disease detection. Continued research and innovation will be pivotal in unlocking the full potential of these cutting-edge biosensors, paving the way for a new stage of advanced diagnostics and personalized healthcare solutions.

## Figures and Tables

**Table 1:** Examples of metal-based nanocomposites used as biosensors.

Nanocomposite	Fabrication Technique	Limit of Detection	Sensitivity	Application	Ref.
MoS_2_ nanoflower/Ag	Chemical synthesis MoS_2_/Ag nanocomposite deposited on an Pt electrode	0.06 mM and 0.0056 mM	147.46 and 14.36 μA mM^−1^ cm^−2^	Enzymatic glucose detection	[77]
PPy/Ag	Three-electrode electrochemical cell fabrication: PPy/Ag composite matrix deposited on a glassy carbon electrode	5 μM	-	Water decontamination	[78]
AgNPs–Pdop@Gr	Electrochemical reaction: AgNPs integration in Graphene (Gr) coated with polydopamine (Pdop)	4 nM for guanine and 2 nM for adenine	-	Adenine and guanine determination	[79]
Ag/MWCNTs/GC	AgNPs coated multi-walled carbon nanotubes (MWCNTs) dispersed on a glassy carbon electrode (GC)	0.2 mM	-	Medical industry	[80]
Ag–S–Zn–O/Indium tin oxide (ITO)	Electrophoretic deposition	0.54 mM	12.56 μA mM^−1^ cm^−2^	Urea detection	[81]
Ag/PMMA	Solution casting and sonication	0.1 mM	41 µA mM^−1 ^ cm^−2^	Non-enzymatic glucose detection	[82]
TiO_2_-modified ZnO nanotubes	Hydrothermal method	0.5 μM	-	Non-enzymatic glucose detection	[83]
rGO/Ag/cotton or polyester	Electron-beam irradiation	9.73 nM for cotton biosensors and 3.05 nM for polyester biosensors	0.0165 mA/cm^2^ for cotton biosensors and 0.0129 mA/cm^2^ for polyester biosensor	Adrenaline detection	[84]
rGO/Au	Hydrothermal reflux	1.73 pM	-	miRNA-122 detection	[85]
ChOx/HRP/AuNPs/APTES/ITO	Cholesterol oxidase (ChOx) and horseradish peroxidase (HRP) immobilization on AuNPs-functionalized ITO electrode	0.235 mg/dL	7.5 µA mg dL^−1^ cm^−2^	Cholesterol detection	[86]
ChOx/PAni–Au–Chitosan/ITO	ChOx-chitosan immobilization on AuNPs-functionalized ITO electrode	37.89 mg/dL	0.86 μA mg dL^−1^	Cholesterol detection	[87]
g-C_3_N_4_/Au	Laser ablation method	140 ppm of methanol	-	Hazardous gases detection	[88]
ZnO/AuNPs	Electrodeposition of ZnO/AuNPs on a glassy carbon electrode	1.8 pM	-	DNA biosensor for Mycobacterium tuberculosis detection	[89]
SiSG-TYR/Fe_3_O_4_-MWCNTs/GCE	Electrochemical synthesis: Fe_3_O_4_-MWCNTs combination together with tyrosinase (TYR), and silica sol–gel (SiSG) deposited on an glassy carbon electrode (GCE)	0.055 μM	-	Catechol and hydroquinone detection from local water	[90]
Cu–FeNPs/Zeolite-A/Graphene	Sol–gel spin coating	0.058 μM	1.97 μA μM^−1^ cm^−2^	Dopamine detection	[91]
